# Artificial intelligence techniques in liver cancer

**DOI:** 10.3389/fonc.2024.1415859

**Published:** 2024-09-03

**Authors:** Lulu Wang, Mostafa Fatemi, Azra Alizad

**Affiliations:** ^1^ Department of Engineering, School of Technology, Reykjavık University, Reykjavík, Iceland; ^2^ Department of Physiology and Biomedical Engineering, Mayo Clinic College of Medicine and Science, Rochester, MN, United States; ^3^ Department of Radiology, Mayo Clinic College of Medicine and Science, Rochester, MN, United States

**Keywords:** artificial intelligence, deep learning, machine learning, liver cancer, hepatocellular carcinoma, medical imaging, diagnosis, prediction

## Abstract

Hepatocellular Carcinoma (HCC), the most common primary liver cancer, is a significant contributor to worldwide cancer-related deaths. Various medical imaging techniques, including computed tomography, magnetic resonance imaging, and ultrasound, play a crucial role in accurately evaluating HCC and formulating effective treatment plans. Artificial Intelligence (AI) technologies have demonstrated potential in supporting physicians by providing more accurate and consistent medical diagnoses. Recent advancements have led to the development of AI-based multi-modal prediction systems. These systems integrate medical imaging with other modalities, such as electronic health record reports and clinical parameters, to enhance the accuracy of predicting biological characteristics and prognosis, including those associated with HCC. These multi-modal prediction systems pave the way for predicting the response to transarterial chemoembolization and microvascular invasion treatments and can assist clinicians in identifying the optimal patients with HCC who could benefit from interventional therapy. This paper provides an overview of the latest AI-based medical imaging models developed for diagnosing and predicting HCC. It also explores the challenges and potential future directions related to the clinical application of AI techniques.

## 1 Introduction

Hepatocellular Carcinoma (HCC), the most common primary liver malignancy, is linked to high mortality rates and stands as a leading cause of cancer-related deaths worldwide (1). Accurate diagnosis and staging of HCC are crucial for improving patient survival rates and treatment outcomes. However, early diagnosis of HCC presents a significant challenge, especially for individuals with chronic liver disease. A notable characteristic of liver cancer is its strong association with liver fibrosis, with over 80% of hepatocellular carcinomas (HCCs) developing in fibrotic or cirrhotic livers (2). This indicates that liver fibrosis plays a vital role in the liver’s premalignant environment.

Medical imaging techniques, including Computed Tomography (CT), Magnetic Resonance Imaging (MRI), and Ultrasound (US), play an essential role in the diagnosis and staging of HCC, supplementing clinical findings, biological markers, and blood tests. CT scans provide detailed cross-sectional images of the liver, aiding in the identification and characterization of tumors (3). MRI offers superior soft tissue contrast, making it invaluable for assessing the extent of liver cancer (4). US, a non-invasive and cost-effective imaging modality, can detect liver tumors by generating liver images using sound waves (5). However, each of these imaging methods has its limitations. For instance, CT scans expose patients to ionizing radiation, potentially heightening the risk of radiation-induced cancer. Moreover, CT scans can be expensive and less accessible in certain healthcare settings. While MRI can produce high-quality images, it can be time-consuming and may not be suitable for patients with claustrophobia or those with metal implants. US has limitations in image quality, particularly in patients with obesity or excessive intestinal gas. Recently, advanced MRI techniques, such as MR Elastography (MRE) and gadoxetic acid-enhanced MRI, have been introduced for liver imaging. These techniques provide high-resolution images without the harmful effects of radiation (6). MRE measures the stiffness of liver tissue, which can assist in differentiating between benign and malignant liver tumors. Gadoxetic acid-enhanced MRI offers dynamic imaging of the liver and can enhance the detection and characterization of HCC.

Diagnosing HCC poses significant challenges. These challenges arise from the prevalence of typical radiological features that are common to other liver tumors or benign conditions. Such similarities in imaging characteristics can lead to misdiagnosis or delayed diagnosis. As a result, patients with liver lesions exhibiting these typical features may require histological confirmation or rigorous monitoring to ensure accurate diagnosis and appropriate treatment.

In recent years, the potential of Artificial Intelligence (AI) techniques in diagnosing HCC has been the subject of extensive research. These techniques have been explored for various purposes such as detecting and evaluating HCC, facilitating treatment, and predicting treatment response (7–13). Numerous studies have investigated the use of AI models in conjunction with different modalities, including electronic health record (EHR) reports, clinical parameters, biological markers, and blood test results, for diagnosing liver cancer (14, 15). AI techniques have emerged as powerful tools capable of extracting valuable insights from voluminous EHRs and developing multimodal AI methods. These methods provide a more comprehensive and accurate depiction of the liver’s internal structure and function.

While many researchers have shown interest in exploring the potential of AI techniques in liver cancer research, there remains a gap in comprehensively evaluating the implementation of single-modal and multi-modal AI techniques for diagnosing HCC. This study aims to bridge this gap by providing a comprehensive review of the most recently developed AI-based techniques that utilize both single and multi-modal data for diagnosing HCC. AI-based techniques hold the potential to enhance early diagnosis, improve diagnostic accuracy, and improve treatment outcomes for patients with HCC. This pivotal area of research could lead to significant advancements in liver cancer diagnosis and prediction.

## 2 Methodology and materials

This research explores the application of AI methodologies in diagnosing and prognosticating primary liver cancer, specifically HCC. The objective is to encapsulate the latest and most relevant discoveries in this rapidly evolving field.

A thorough literature review was conducted using databases such as PubMed, Scopus, Semantic Scholar, IEEEXplore, and Web of Science, up until March 31, 2024. During this process, several key terms such as “artificial intelligence”, “deep learning”, “machine learning”, “liver cancer”, “hepatocellular carcinoma”, “multi-modal”, “medical imaging”, “US”, “CT”, and “MRI” were searched in the title and/or abstract or all field. References from relevant articles were examined to identify additional qualifying publications.

An expert review of the eligible literature was carried out, and the most informative and pertinent citations were chosen for inclusion. The studies selected were those that integrated AI techniques with medical imaging datasets, including US, CT, and MRI, in conjunction with Electronic Health Records (EHR) and clinical parameters. Studies that did not utilize medical imaging techniques or AI models specifically targeting primary liver cancer were excluded.

The search was confined to peer-reviewed articles, conference proceedings, dissertations, and book chapters published in English from January 2010 to March 2024. These publications were retrieved, screened, and reviewed by the authors. One researcher then undertook the data extraction, focusing on the methods and results of each study.

**Figure 1:**
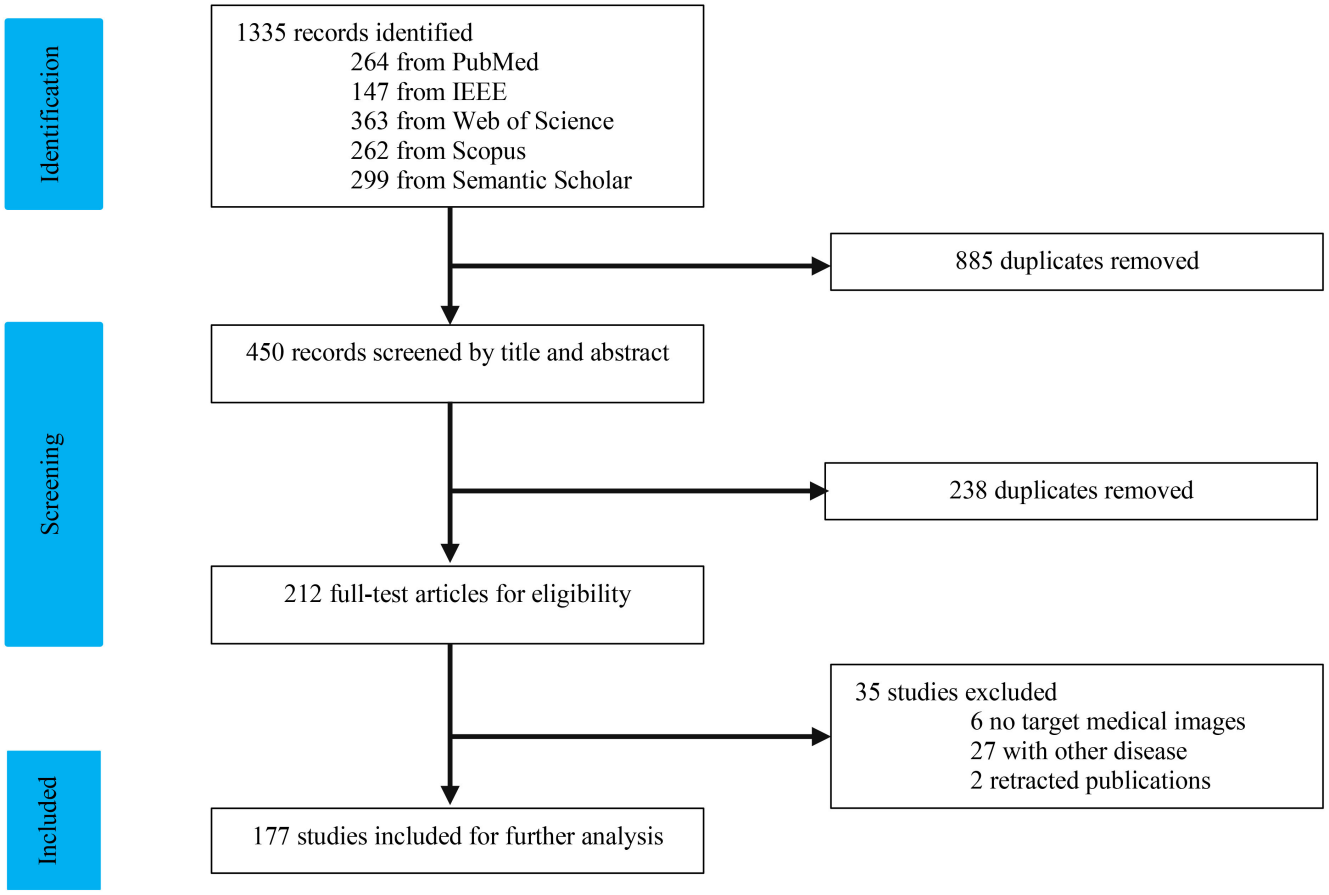
Flowchart of study selection.

As depicted in **Figure 1**, our study selection process began with 1334 records. After removing 885 duplicates, we screened 450 records. The title and abstract screening led to the exclusion of 240 studies, leaving 210 for full-text review. Following a comprehensive evaluation, 177 articles (7, 16–186) were deemed suitable for this study. We categorized the modalities into four groups: US (n = 34), CT (n = 95), MRI (n = 34), and multi-modal (n=19). The characteristics of the included studies are detailed in **Tables 1**–**10**.

**Table 1:** AI-based US approaches for HCC diagnosis.

Ref	Year	AI Model	US Method	Task	Dataset	AUC	Accuracy	Sensitivity	Specificity
(17)	2010	FSVM	B-mode US	Classify benign and malignant liver lesions	200 images	0.984	0.97	1	0.955
(17)	2010	FSVM	B-mode US	Classify benign and malignant liver lesions	450 images	0.971	0.951	0.92	0.955
(18)	2011	Two-step neural network	B-mode US	Classify FLLs	111 images (88 patients)	~	0.864	~	~
(18)	2011	Two-step neural network	B-mode US	Detect FLLs	111 images (88 patients)	~	0.903	~	~
(7)	2014	NNE	B-mode US	Diagnosis of FLLs	108 patients	~	0.95	~	~
(19)	2015	ANN	B-mode US	Diagnosis of FLLs	115 patients	~	>0.96	~	~
(20)	2017	ANN	B-mode US	Diagnosis of FLLs	110 images	~	0.972	0.98	0.957
(21)	2018	SVM	B-mode US	Classify benign and malignant liver lesions	189 images (94 patients)	~	0.966	0.969	0.998
(22)	2019	Supervised DL	B-mode US	Detection and characterization of FLLs as benign and malignant	Training set: 367 images (367 patients), Test set: 177 patients	Training: mean ACU:0.935 for detection, mean ACU: 0.916 for characterization, Test: mean ACU: 0.891 for detection	~	~	~
(23)	2020	CNN	B-mode US	Characterization of FLLSs as benign or malignant	Training: 16500 images (1446 Patients), Internal validation: 4125 images (369 patients), External validation: 3718 images (328 patients)	Training: mean ACU: 0.765~0.925 Internal validation: mean ACU: 0.859~0.966 External validation: mean ACU: 0.750~0.924	~	~	~
(24)	2020	CNN	B-mode US	Differentiate HCC and PAR	GE9 dataset	0.91	0.8484	0.8679	0.8295
(24)	2020	CNN	B-mode US	Differentiate HCC and PAR	GE7 dataset	0.95	0.91	0.9437	0.8838
(25)	2021	LR, k-NN, MLP, RF, SVM	B-mode US	Characterization of FLLSs as benign or malignant	114 patients Training: 91, Test:23	Mean AUC: 0.737~0.816	Mean accuracy: 0.729~0.843	~	~
(26)	2021	DL	B-mode US	Diagnosis of FLLs	4309 images (3873 patients)	0.947	0.822	0.867	0.987
(27)	2021	CNN	B-mode US	Diagnosis of FLLs	40397 images (3847 patients)	~	0.949	0.736	0.978
(28)	2021	CNN	B-mode US	Classify benign and malignant liver lesions	911 images (596 patients)	0.860	0.84	0.87	0.78
(29)	2021	CNN	Endoscopic US	Classify benign and malignant liver lesions	210685 images (256 patients)	0.861 (image), 0.904 (video)	~	0.9(image), 1 (video)	0.71(image), 0.80(video)
(30)	2021	SVM	B-mode US	Differentiate HCC and ICC	226 patients, Training: 149 Test: 38 External validation: 39	Training: 0.840~0.975, Test: 0.711~0.936, External validation: 0.730~0.874	Training: 0.7047~0.8926, Test:: 0.7105~0.8684, External validation: 0.6923 ~0.8718	Training: 0.7742~0.9677, Test: 0.7~0.9, External validation: 0.6667~0.8887	Training: 0.6864 ~0.8729, Test: 0.7143 ~0.8571, External validation: 0.6667~0.8667
(31)	2021	SVM	B-mode US	Prediction of pathological grading of HCC	193 patients Training: 128 Test: 32 External validation: 33	Training: 0.788~0.977, Test: 0.72~0.874, External validation: 0.77~0.849	Training: 0.7422~0.9219, Test:: 0.6875~0.8438, External validation: 0.6667 ~0.8182	Training: 0.6471~0.902, Test: 0.5714~0.8571, External validation: 0.75	Training: 0.8052 ~0.9351, Test: 0.72 ~0.84, External validation: 0.619~0.8571
(32)	2022	CNN	B-mode US	Diagnosis of FLLs	70950 images	~	0.934	0.675	0.96
(33)	2022	DL	B-mode US	Diagnosis of HCC	407 patients	0.936	0.864	0.96	0.769
(34)	2022	ResNet18	B-mode US	Differentiate and predict HCC	513 patients	0.855(training), 0.709 (validation)	~	~	~
(35)	2023	CNN	Quantitative US	Diagnosis of hepatic steatosis	173 patients	0.97	~	0.90	0.91
(36)	2012	ANN	CEUS	Diagnosis of FLLs	112 patients	~	0.9442	0.932	0.897
(37)	2014	DL	CEUS	Diagnosis of FLLs	22 patients	~	0.8636	0.8333	0.8750
(38)	2015	SVM	CEUS	Diagnosis of FLLs	52 video sequences	~	0.903	0.931	0.869
(39)	2017	SVM	CEUS	Classify benign and malignant liver lesions	98 patients		0.918	0.94	87.1
(40)	2018	DCCA -MKL	CEUS	Classify benign and malignant liver lesions	93 patients	0.953	0.9041	0.9356	0.8689
(41)	2018	ANN	CEUS	Differentiating benign from malignant liver lesions	106 lesions	0.829~0.883	0.80~0.811		
(42)	2019	3D CNN	CEUS	Classify aHCC and FNH	4420 images	~	0.931	0.945	0.936
(43)	2020	SVM	CEUS	Differentiation between aHCC and FNH	257 images	0.944	~	0.9476	0.9362
(44)	2021	DL	CEUS	Classify five types of FLLs	273 video files (91 patients)	~	0.88	~	~
(45)	2021	CNN	CEUS	Classify benign and malignant liver lesions	363 patients	0.934	0.91	0.927	0.851
(46)	2021	SVM	CEUS	Preoperative histological grading	235 HCC lesions: 65 high grade and 170 low grade lesions	0.665~0.785	~	~	~
(47)	2022	ML	CEUS	Classify benign and malignant liver lesions	87 images (72 patients)	0.840	0.84	0.76	0.92
(48)	2024	CNN-LSTM	CEUS	Classify benign and malignant liver lesions	440 patients	0.91	~	0.95	0.7
(48)	2024	3D-CNN	CEUS	Classify benign and malignant liver lesions	440 patients	0.88	~	0.96	0.55
(48)	2024	ML-TIC	CEUS	Classify benign and malignant liver lesions	440 patients	0.78	~	0.96	0.21

aHCC, a typical HCC; AUC, area under the curve; CNN, convolutional neural network; DCCA –MKL, deep canonical correlation analysis and multiple kernel learning; DL, deep learning; FNH, focal nodular hyperplasia; HCC, hepatocellular carcinoma; iANN, improved artificial neural network; ML- TIC, machine learning based time-intensity curve; NNE, neural network ensemble; PAR, cirrhotic parenchyma; SVM, support vector machine; US, ultrasound.

**Table 2:** AI-driven CT models for segmentation of liver and liver tumors.

Ref	Year	AI Model	Imaging method	Task	Dataset	Results
(49)	2015	Model based Shape Constraints and Deformable Graph Cut	CT	Liver segmentation	3DIRCADb	VOE=9.15
(49)	2015	Model based Shape Constraints and Deformable Graph Cut	CT	Liver segmentation	Sliver07	VOE=62.4
(53)	2017	CNN + MRFs	CT	Liver segmentation	Hospital dataset	Dice= 0.83
(54)	2017	U-Net	CT	Liver segmentation	3DIRCADb	Dice=0.923, VOE=14.21
(55)	2018	Faster R-CNN	CT	Liver segmentation	SLIVER07	VOE = 5.06,VD = 0.09
(55)	2018	Faster R-CNN	CT	Liver segmentation	3DIRCADb	VOE = 0.0867, VD = 0.57
(56)	2018	V-net	CT	Liver segmentation	3DIRCADb	Dice=0.874, VOE=21.85
(56)	2018	V-net	CT	Liver segmentation	SLIVER07	Dice=0.872, VOE=21.15
(57)	2018	H Dense UNet	CT	Liver segmentation	3DIRCADb	Dice=0.930, VOE=12.87
(57)	2018	H Dense UNet	CT	Liver segmentation	SLIVER07	Dice=0.927, VOE=13.29
(58)	2018	U-net+ GAN	CT	Liver segmentation	3DIRCADb	Dice= 0.94
(59)	2019	Channel-UNet	CT	Liver segmentation	3DIRCADb	Dice= 0.984
(60)	2020	BS U-Net	CT	Liver segmentation	LiTS	Dice= 0.961
(61)	2020	RA U-Net	CT	Liver segmentation	3DIRCADb	Dice= 0.830, VOE = 4.5
(61)	2020	RA U-Net	CT	Liver segmentation	LiTS	Dice= 0.961, VOE = 7.4
(62)	2020	Multi-Layer U-Net	CT	Liver segmentation	3DIRCADb	Dice = 0.9645
(62)	2020	Multi-Layer U-Net	CT	Liver segmentation	LiTS	Dice = 0.9638
(63)	2020	3DResUNet	CT	Liver segmentation	3DIRCADb	Dice = 0.958
(64)	2020	CNN	CT	Liver segmentation	Hospital dataset	Dice = 0.949
(65)	2020	BATA-Unet	CT	Liver segmentation	MICCAI	Dice=0.9788, VOE=4.5, RVD=0.04%, ASD=0.05mm, MSD=0.08mm
(65)	2020	BATA-Unet	CT	Liver segmentation	3DIRCAD	Dice=0.9671, VOE=0.115, RVD=0.08%, ASD=0.14mm, MSD=0.16mm
(66)	2021	Multi Res U-Net	CT	Liver segmentation	3DIRCADb	Dice= 0.88
(67)	2021	DenseXNet	CT	Liver segmentation	3DIRCADb	Dice= 0.968
(67)	2021	DenseXNet	CT	Liver segmentation	LiTS	Dice= 0.9668
(68)	2021	T3scGAN	CT	Liver segmentation	LiTS	Dice= 0.961
(69)	2021	2.5D light-weight nnU-Net	CT	Liver segmentation	LiTS	Dice= 0.962
(70)	2021	2.5D U-Net	CT	Liver segmentation	LiTS	Dice= 0.928
(71)	2021	2.5D P U-Net	CT	Liver segmentation	LiTS	Dice= 0.962
(72)	2021	DFS U-Net	CT	Liver segmentation	LiTS	Dice= 0.949
(73)	2021	MSN-Net	CT	Liver segmentation	LiTS	Dice= 0.942
(74)	2021	U-Net	CT	Liver segmentation	LiTS	Dice=0.9693 for training, Dice=0.9077 for validation, Dice=0.9084 for testing
(75)	2022	Casecade DL	CT	Liver segmentation	LiTS	Dice= 0.9564, VOE=0.0828
(76)	2022	PADLLS	CT	Liver segmentation	SLIVER07	Dice= 0.957, VOE=0.0814
(76)	2022	PADLLS	CT	Liver segmentation	3DIRCADb	Dice= 0.965, VOE=0.0666
(77)	2022	DALU-Net	CT	Liver segmentation	Custom	Dice=0.899
(78)	2022	nnU-Net	CT	Liver segmentation	LiTS-IRCAD	global Dice=0.974,
(79)	2023	SLIC-DGN	CT	Liver segmentation	LiTS17	Acc=0.991, Dice=0.911, Mean IoU=0.908, Sen= 0.994, Recall=0.994, Prec=0.912
(80)	2023	DD-UDA	multi-phase CT	Liver segmentation	LiTS & MPCT-FLLs	IoU=0.823 (PV), IoU=0.811 (ART), IoU=0.800 (NC)
(81)	2023	RMAU-Net	CT	Liver segmentation	LiTS	Dice=0.9552
(81)	2023	RMAU-Net	CT	Liver segmentation	3D-IRCABb	Dice=0.9697
(82)	2023	AIM-Unet	CT	Liver segmentation	CHAOS	Dice=0.9786, Jac=0.9610
(82)	2023	AIM-Unet	PET/CT	Liver segmentation	Clinical data	Dice=0.9738, Jac=0.9495
(83)	2023	MAD-UNet	CT	Liver segmentation	LiTS17	Dice=0.9727
(83)	2023	MAD-UNet	CT	Liver segmentation	Sliver07	Dice=0.9752
(83)	2023	MAD-UNet	CT	Liver segmentation	3DIRCADb	Dice=0.9691
(84)	2023	Eres-UNet++	CT	Liver segmentation	LiTS	Acc=0.958, IoU=0.921, F1-Score=0.959, Recall=0.96
(85)	2023	Dual-path Network with Swin Transformer Encoding	CT	Liver segmentation	LiTS	Dice=0.962
(86)	2024	Spider-UNet	CT	Liver segmentation	LiTS17& 2018 MICCAI	Dice= 0.459
(86)	2024	3D UNet	CT	Liver segmentation	LiTS17& 2018 MICCAI	Dice= 0.54
(86)	2024	V-Net	CT	Liver segmentation	LiTS17& 2018 MICCAI	Dice= 0.57
(86)	2024	FCN-RNN	CT	Liver segmentation	LiTS17& 2018 MICCAI	Dice= 0.58
(86)	2024	LSTM-Unet	CT	Liver segmentation	LiTS17& 2018 MICCAI	Dice=0.59
(86)	2024	3DRes-Unet	CT	Liver segmentation	LiTS17& 2018 MICCAI	Dice= 0.62
(86)	2024	MP-UNet	CT	Liver segmentation	LiTS17& 2018 MICCAI	Dice= 0.625
(86)	2024	3D VGN	CT	Liver segmentation	LiTS17& 2018 MICCAI	Dice= 0.649
(86)	2024	UMCT	CT	Liver segmentation	LiTS17& 2018 MICCAI	Dice= 0.65
(86)	2024	nnU-Net	CT	Liver segmentation	LiTS17& 2018 MICCAI	Dice= 0.675
(86)	2024	3D-GCCN	CT	Liver segmentation	LiTS17& 2018 MICCAI	Dice= 0.70
(86)	2024	Improved V-Net	CT	Liver segmentation	LiTS17& 2018 MICCAI	Dice= 0.7253
(87)	2024	SADSNet	CT	Liver segmentation	LITS	Dice= 0.9703
(87)	2024	SADSNet	CT	Liver segmentation	3DIRCADb	Dice= 0.9611
(87)	2024	SADSNet	CT	Liver segmentation	SLIVER	Dice= 0.9740
(88)	2024	SD-Net	CT	Liver segmentation	LiTS	Dice>0.94
(89)	2024	LRENet	CT	Liver segmentation	LiTS, 3Dircadb01 & Clinical data	Acc=0.9769, IoU=0.8608, Dice=0.9252
(49)	2015	CNN	phase enhanced CT	Liver tumor segmentation	26 images	Prec=0.867
(90)	2016	End-to-end 3D FCN with CRF	CT	Liver tumor segmentation	SLIVER07	VOE =5.42, VD =1.75
(51)	2017	FCN	CT	Liver tumor segmentation	2 databases Training: 3809 images	VOE =15.6~38.2, 8.1~19.1 for each dataset
(50)	2017	CNN	CT	Liver tumor detection and segmentation	246 tumors (97 new tumors)	True positive rate =0.72~0.86 for detection
(57)	2018	H Dense UNet	CT	Liver tumor segmentation	3DIRCADb & LiTS	Dice =0.824
(91)	2018	FCN	CT	Liver tumor segmentation	Clinical data	True positive rate=0.964
(92)	2018	ResNet based SSD	CT	Liver tumor segmentation	Clinical data	Prec =0.533
(93)	2019	Nested U-Net	CT	Liver tumor segmentation	LiTS	Pixel accuracy =0.9997, IoU =0.7917, Rand Index=0.9106
(59)	2019	Channel-UNet	CT	Liver tumor segmentation	3DIRCADb	Dice =0.940
(94)	2019	3D Residual U-Net	CT	Liver tumor segmentation	109 volumes	Dice =0.69, Sen= 0.682
(60)	2020	BS U-Net	CT	Liver tumor segmentation	LiTS	Dice =0.569
(61)	2020	RA U-Net	CT	Liver tumor segmentation	3DIRCADb	Dice =0.977, VOE =25.5
(61)	2020	RA U-Net	CT	Liver tumor segmentation	LiTS	Dice =0.595, VOE =38.9
(62)	2020	Multi-Layer U-Net	CT	Liver tumor segmentation	3DIRCADb	Dice =0.7334
(62)	2020	Multi-Layer U-Net	CT	Liver tumor segmentation	LiTS	Dice =0.7369
(95)	2020	SegNet	CT	Liver tumor segmentation	3DIRCADb	Dice =0.9522
(96)	2020	Modified SegNet	CT	Liver tumor segmentation	3DIRCADb	True positive rate= 0.988
(67)	2021	DenseXNet	CT	Liver tumor segmentation	3DIRCADb	Dice =0.764
(67)	2021	DenseXNet	CT	Liver tumor segmentation	LiTS	Dice =0.6911
(70)	2021	2.5D U-Net	CT	Liver tumor segmentation	LiTS	Dice =0.672
(71)	2021	2.5D P U-Net	CT	Liver tumor segmentation	LiTS	Dice =0.735
(68)	2021	CGBS-Net	CT	Liver tumor segmentation	Hospital dataset	Dice =0.9641
(45)	2022	TransNUNet	CT	Liver tumor segmentation	LiTS	Dice =0.9793 (training), Dice=0.9196 (testing)
(45)	2022	TransUNet	CT	Liver tumor segmentation	LiTS	Dice=0.9456 (training), Dice=0.8713 (testing)
(45)	2022	UNet	CT	Liver tumor segmentation	LiTS	Dice=0.8619 (training), Dice=0.7185 (testing)
(45)	2022	UNet3+	CT	Liver tumor segmentation	LiTS	Dice=0.9531 (training), Dice=0.8261 (testing)
(97)	2023	MANet	CT	Liver tumor segmentation	3DIRCADb	Dice=0.64, IoU =0.5227, Acc =0.9947, Sen =0.624, Spec =0.999,VOE =0.4773
(97)	2023	MANet	CT	Liver tumor segmentation	LiTS	Dice=0.8145, IoU =0.7084, Acc =0.9947, Sen =0.8723, Spec =0.997, VOE =29.15
(79)	2023	SLIC-DGN	CT	Liver tumor segmentation	LiTS17	Dice=0.9, IoU =0.892, Acc =0.987, Sen =0.979, Spec =0.887
(98)	2023	Three-path structure with MSFF, MFF, EI, and EG	CT	Liver tumor segmentation	LiTS17	Dice=0.8555, IoU =0.9045, Acc =0.9979, Sen =0.8682, Spec =0.9993
(99)	2023	En–DeNet	CT	Liver tumor segmentation	3DIRCADb	Dice=0.8481, Acc =0.8808, Prec =0.8613
(99)	2023	En–DeNet	CT	Liver tumor segmentation	LiTS	Dice=0.8594, Acc =0.9217, Prec =0.894
(84)	2023	Eres-UNet++	CT	Liver tumor segmentation	LiTS	IoU =0.84, Acc =0.893, F1 score =0.913
(85)	2023	Dual-path Network with Swin Transformer Encoding	CT	Liver tumor segmentation	LiTS	Dice=0.681
(100)	2023	Enhanced M-RCNN	CT	Liver tumor segmentation	LiTS17	Dice=0.957, VOE =9.5
(100)	2023	Enhanced M-RCNN	CT	Liver tumor segmentation	Sliver07	Dice=0.9731, VOE =5.37
(82)	2023	AIM-Unet	CT	Liver tumor segmentation	LiTS	Dice=0.756
(82)	2023	AIM-Unet	CT	Liver tumor segmentation	3DIRCADb	Dice=0.655
(81)	2023	RMAU-Net	CT	Liver tumor segmentation	LiTS	Dice=0.7616
(81)	2023	RMAU-Net	CT	Liver tumor segmentation	3DIRCADb	Dice=0.8307
(87)	2024	SADSNet	CT	Liver tumor segmentation	LiTS	Dice=0.8781
(87)	2024	SADSNet	CT	Liver tumor segmentation	3DIRCADb	Dice=0.8750
(101)	2024	SEU2-Net	CT	Liver tumor segmentation	PUFH	Dice=0.9504, IoU =0.9055, Acc =0.997
(101)	2024	SEU2-Net	CT	Liver tumor segmentation	LiTS	Dice=0.9093, IoU =0.8337, Acc =0.9986
(89)	2024	LRENet	CT	Liver tumor segmentation	LiTS, 3Dircadb01, Clinical data	Dice=0.7312, IoU =0.5763, Acc =0.7548
(102)	2024	DS-HPSNet	CT	Liver tumor segmentation	3Dircadb1	Dice=0.815, Sen =0.807, Prec =0.83
(102)	2024	DS-HPSNet	CT	Liver tumor segmentation	MSD	Dice=0.749, Sen =0.726, Prec =0.762
(64)	2020	CNN	CECT	Liver segmentation	Clinical data	Dice= 0.961
(103)	2022	CNN	CECT	Liver tumor segmentation	58 patients	Dice=0.987, Prec =0.967
(104)	2023	3D UNet	CECT	Liver segmentation	170 patients	Best Dice=0.95
(105)	2023	U-net	CECT	Liver segmentation	259 patients	Dice=0.96
(105)	2023	U-net	CECT	Liver tumor segmentation	259 patients	Dice=0.86

3DIRCADb, 3D Image Reconstruction for Comparison of Algorithm Database; 3DIRCADb01, 3D Image Rebuilding for Comparison of Algorithms Database; Acc, accuracy; AUC, area under the curve; CECT, Contrast-enhanced CT; DD-UDA, dual discriminator-based unsupervised domain adaptation; DS-HPSNet: Dual-stream Hepatic Portal Vein segmentation Network; EG, edge-guiding; EI, edge-inspiring; En–DeNet, Encoder–Decoder Network; FCN, fully convolutional network; CNN, convolutional neural networks; HFSNet, hierarchical fusion strategy of deep learning networks; IoU, intersection over union; LiTS, liver tumor segmentation; LiTS17, liver tumor segmentation 2017; LRENet, location-related enhancement network; MAD-UNet, multi-scale attention and deep supervision-based 3D UNet; MCC, Matthews’s correlation coefficient; MFF, multi-channel feature fusion; MRFs, Markov random fields; MSD, medical segmentation decathlon hepatic vessel segmentation dataset; MSFF, multi-scale selective feature fusion; PADLLS, pipeline for automated deep learning liver segmentation; Prec, precision; RD DLIR-H, high-strength deep learning image reconstruction; RD DLIR-M, medium-strength deep learning image reconstruction; RMAU-Net, residual multi-scale attention U-Net; VOE, Volume overlap error; SD-Net, semi-supervised double-cooperative network; Sen, sensitivity; SLIC-DGN, SLIC-based deep graph network; Spec, specificity; VOE, volume overlap error.

**Table 3:** AI-based CT models for diagnosing HCC.

Ref	Year	AI Model	Tasks	Imaging method	Dataset	Results
(110)	2018	CNN	Characterization of liver lesions: classification in five categories, and malignant (HCC and non-HCC liver cancers) vs indeterminate and benign lesions (hemangiomas and cysts)	Three-phase CT	Training: 460 patients Testing: 100 patients	Acc =0.84, AUC =0.92 Classification: Training: Median Acc=0.95~0.97, Testing: Median Acc=0.48−0.84, Sen=0.11~1, Malignant vs the rest: Testing: Median AUC=0.61~0.92
(111)	2018	Mics-CNN	Detect FLLs	Multi-phase CT	89 patients	F1 score =0.82
(91)	2018	FCN	Detect liver metastases	CT	20 patients	Acc =0.946
(106)	2019	ML	Distinguish HCC from non-HCC lesions in cirrhotic patients	CT	13920 images (178 patients)	AUC =0.81 for training set, AUC=0.66 for external validation set
(107)	2019	SVM, k-NN, Ensemble classifier	Characterization of FLLs as malignant or benign	CT	179 patients: 98 benign and 81 malignant lesions	Acc=0.966~0.983, Spec=0.9423~0.9703 for HCC
(111)	2019	CNN	Characterization of FLLs (five categories)	CT	89 patients	Sen=0.79~1
(52)	2019	DNN	Classify HEM, HCC and MET	CT	225 images	Acc =0.9939, Sen=1, Spec=0.9909
(108)	2020	ANN, SVM, CNN	Classification of nodular, diffuse and massive HCC	CT	165 images: 46 diffuse tumors, 43 nodular tumors And 76 massive tumors	Average AUC=0.957~0.990, Average Acc=0.926~0.984 (average values for all three models)
(109)	2020	MP-CDN (3 models)	Detect HCC from other FLLs	Multi-phase CT	342 patients with 449 lesions (194 HCC), Training set: 359 lesions Test set: 90 lesions	Acc=0.811~0.856, AUC=0.862~0.925, Sen=0.744~0.923, Spec=0.725~0.941
(113)	2020	CNN, SVM	Differentiation between HCC and ICC	Multi-phase CT	187 HCC and 70 ICC lesions	Acc =0.88, TPR=0.9518 for HCC, TPR=0.6944 for ICC
(25)	2020	Radiomics eXtreme Gradient Boosting	Grading of HCC	CT	Training: 237 Patients, Testing: 60 patients	Training: AUC=0.6915~0.9964,Acc=0. 6118~0.9705, Sen=0.6067~0.9551, Spec=0.5135~0.8041, Testing: AUC=0.6128~0.8014, Acc=0.483~0.7, Sen=0.4348~0.6522, Spec=0.3784~0.8108
(116)	2020	CNN	Detect liver cancer in hepatitis patients	CT	NHIRD	Acc =0.98, Sen =0.783, Spec =0.990, Prec =0.793, F1 score =0.788, MCC =0.777, AUC =0.886
(116)	2020	SVM	Detect liver cancer in hepatitis patients	CT	NHIRD	Acc =0.961,Sen =0.343,Spec =0.987, Prec =0.533, F1 score =0.417, MCC =0.409, AUC =0.665
(116)	2020	RNN	Detect liver cancer in hepatitis patients	CT	NHIRD	Acc =0.945,Sen =0.357,Spec =0.969, Prec =0.329, F1 score =0.342, MCC =0.314, AUC =0.945
(116)	2020	LSTM	Detect liver cancer in hepatitis patients	CT	NHIRD	Acc =0.936, Sen =0.349, Spec =0.967, Prec =0.353, F1 score =0.351, MCC =0.317, AUC =0.936
(116)	2020	GRU	Detect liver cancer in hepatitis patients	CT	NHIRD	Acc =0.960, Sen =0.529, Spec =0.978, Prec =0.500, F1 score =0.514, MCC =0.493, AUC =0.960
(112)	2021	multi-modality and multi-scale CNN	Characterization of FLLs: malignant (HCC, ICC and metastasis) versus benign lesions (cyst, hemangioma, and FNH), classification of FLLs (Six-class)	CT	616 FLLs	Detection: Average Prec=0.828, Classification: Binary classification: Acc=0.825, AUC=0.921, Sen =0.766~0.884, Spec=0.766~0.884, Six-class classification: Acc=0.734, AUC=0.766~0.983, Sen =0.466~0.931, Spec=0.919~0.986
(117)	2021	HCCNet	Detect HCC	CT	7512 patients, Internal test: 385, External test: 556	Internal testing: Acc =0.81, Sen =0.784, Spec =0.844, F1 score =0.824, External testing: Acc =0.813, Sen =0.894, Spec =0.74, F1 score =0.819
(118)	2021	STIC	Classify HCC and ICC	CT	723 patients	Acc =0.862, AUC =0.893
(118)	2021	STIC	Detect malignant hepatic tumors	CT	723 patients	Acc =0.726
(119)	2021	MDL-CNN	Detect HCC, hepatic cysts, MET, HEM	CT	4212 images	Dice =0.957
(119)	2021	MDL-CNN	Classify HCC, hepatic cysts, MET, HEM	CT	4212 images	Dice =0.9878
(120)	2021	multi-scale CNN	Detect hepatic cysts, HEM, MET	CT	1290 images	Acc =0.873
(112)	2021	multi-modality and multi-scale CNN	Detect FLLs, including HCC, ICC, MET, hepatic cysts, HEM, FNH	CT	616 images	Prec =0.828, F1 score =0.878
(112)	2021	multi-modality and multi-scale CNN	Classify FLLs (Binary)	CT	616 images	Acc =0.825
(112)	2021	multi-modality and multi-scale CNN	Classify FLLs (Six-class)	CT	616 images	Acc =0.734
(114)	2021	ML-EM	Detection and classification of malignant liver lesions (HCC and secondary liver lesions)	CT	1638 images	Detection: Acc =0.9839~1, AUC=0.99−1.00 Classification: Acc =0.7638~0.8701, AUC=0.77~0.99
(121)	2021	Mask R-CNN	Detect primary hepatic malignancies in HCC patients	CT	1350 images (1320 patients)	Sen =0.848
(122)	2021	CNN	Diferentiating ICC from HCC	Three-phase CT	617 patients	Acc =0.61, Sen =0.75, Spec =0.88, AUC =0.87
(122)	2021	CNN	Diferentiating pHCC from mHCC	Three-phase CT	617 patients	Acc =0.61, Sen =0.62, Spec =0.68, AUC =0.68
(123)	2022	SVM	Classify HCC, MET, HHs	CT	452 patients	Acc =0.88
(124)	2022	Googlenet	Detect and classify FLLs	CT	3D-IRCADb01	Acc =0.93, F1 score =0.9255, Dice =0.64
(124)	2022	Unet	Detect and classify FLLs	CT	3D-IRCADb01	Acc =0.9865, F1 score =0.9875, Dice =0.83
(124)	2022	Dense 3D	Detect and classify FLLs	CT	3D-IRCADb01	Acc =0.89, Dice =0.94
(124)	2022	Dense-Net	Detect and classify FLLs	CT	3D-IRCADb01	Acc =0.92, F1 score =0.93
(124)	2022	SegNet VGG-16	Detect and classify FLLs	CT	3D-IRCADb01	Acc =0.86
(124)	2022	GMM	Detect and classify FLLs	CT	3D-IRCADb01	Acc =0.9538
(124)	2022	SVM +RF	Detect and classify FLLs	CT	3D-IRCADb01	Acc =0.91
(125)	2023	RD DLIR-M	Detect FLLs	CT	296 patients	Acc =0.8741, Sen =0.749, Spec =0.579
(125)	2023	RD DLIR-H	Detect FLLs	CT	296 patients	Acc =0.7926, Sen =0.625,Spec =0.417
(126)	2023	ML	Detect hepatic	CT	LI-RADS2018	Acc =0.701, Sen =0.67,Spec =0.91
(127)	2023	DL-CB	Detect FLLs	CT	68 patients	Acc =0.733
(127)	2023	DL-CB	Detect HCC	CT	68 patients	Acc =0.704
(115)	2023	Modified Unet-60	Detect and classify FLLs	CT	3Dircadb	Acc =0.9861, Sen =0.9722, Spec =1, Dice =0.9859
(115)	2023	AdaBoost M1	Detect and classify FLLs	CT	3Dircadb	Acc =0.9072, Sen =0.9247, Spec =0.8797
(115)	2023	SVM	Detect and classify FLLs	CT	3Dircadb	Acc =0.9517, Sen =0.9576, Spec =0.9422
(115)	2023	KNN	Detect and classify FLLs	CT	3Dircadb	Acc =0.9387, Sen =0.9531, Spec =0.9256
(115)	2023	Naïve Bayes	Detect and classify FLLs	CT	3Dircadb	Acc =0.9194, Sen =0.9365, Spec =0.8991
(115)	2023	Random forest	Detect and classify FLLs	CT	3Dircadb	Acc =0.9486, Sen =0.9538, Spec =0.9388
(115)	2023	DNN	Detect and classify FLLs	CT	3Dircadb	Acc =0.9838, Sen =0.9909, Spec =1
(115)	2023	ANN	Detect and classify FLLs	CT	3Dircadb	Acc =0.8889, Sen =0.8288,Spec =0.9523
(115)	2023	MLP	Detect and classify FLLs	CT	3Dircadb	Acc =0.8915, Sen =0.8801,Spec =0.9038, Dice =0.8905
(115)	2023	CNN	Detect and classify FLLs	CT	3Dircadb	Acc =0.88
(115)	2023	CNN	Detect and classify FLLs	CT	3Dircadb	Acc =0.96
(115)	2023	CNN	Detect and classify FLLs	CT	3Dircadb	Acc =0.8958
(115)	2023	CNN	Detect and classify FLLs	CT	3Dircadb	Acc =0.869
(115)	2023	KNN, SVM, RF	Detect and classify FLLs	CT	3Dircadb	Acc =0.966
(128)	2024	HFS-Net	Detect HCC	CT	595 patients	Sen =0.843, Prec =0.755, F1 score =0.796, Dice =0.828
(129)	2004	SVM	Detect hypodense hepatic lesions	CECT	56 images (51 patients)	Sen =0.90
(129)	2004	SVM	Classify hypodense hepatic lesions	CECT	56 images (51 patients)	Sen =0.95
(130)	2019	CNN	Classify FNH and HCA	CECT	98 patients	AUC =0.824

3DIRCADb, 3D image reconstruction for comparison of algorithm database; Acc, accuracy; ANN, artificial neural network; AUC,area under the curve; CNN, convolutional neural networks; DCNN, deep convolutional neural networks; DL-CB, deep-learning-based contrast-boosting; DNN, deep neural network; FCN, fully convolutional network; FNH, focal nodular hyperplasia; GRU, gated recurrent unit; HCA, hepatocellular adenoma; HCC, hepatocellular carcinoma; HEM, hemangioma; HFS-Net, hierarchical fusion strategy of deep learning networks; ICC, intrahepatic cholangiocarcinoma; KNN, K-nearest neighbors KNN; LI-RADS2018, liver imaging reporting and data system version 2018; LSTM, long short-term memory; MCC, Matthews’s correlation coefficient; MDL-CNN, multi-channel deep learning CNN; MET, metastatic carcinoma; ML, machine learning; ML-EM, multi-level ensemble model; NHIRD, national health insurance research database; Prec,precision; RD DLIR-H, high-strength deep learning image reconstruction; RD DLIR-M, medium-strength deep learning image reconstruction; RNN, recurrent neural network; Sen, sensitivity; Spec, specificity; SVM, support vector machine.

**Table 4:** AI-based CT models for HCC prognostication.

Ref	Year	AI Model	Tasks	Imaging modality	Dataset	Internal validation Results	External validation Results
(133)	2021	Multi-task DL	Predict future MVI in HCC	CT	366 patients, Training:281, Testing: 85	AUC=0.836	~
(134)	2021	TwinLiverNet	Predict TACE in HCC patients	CT	97 images (92 patients)	Acc=0.825, Sen=0.817, Spec=0.833	~
(134)	2021	LiverNet	Predict TACE in HCC patients	CT	97 images (92 patients)	Acc=0.741, Sen=0.717, Spec=0.767	~
(134)	2021	Baseline Net (no augm)	Predict TACE in HCC patients	CT	97 images (92 patients)	Acc=0.433, Sen=0.40, Spec=0.467	~
(134)	2021	Baseline Net (data augm)	Predict TACE in HCC patients	CT	97 images (92 patients)	Acc=0.567, Sen=0.533, Spec=0.60	~
(131)	2021	cML+DL	Predict TACE in HCC patients	CT	310 patients	AUC=0.994	~
(72)	2021	ResNet-18 (AP)	Predict MVI in HCC patients	CT	309 patients, Training:216, Validation: 93, External testing: 164	Acc=0.68, Sen=0.96, Spec=0.56, AUC=0.82	Acc=0.66, Sen=0.8, Spec=0.62, AUC=0.75
(72)	2021	ResNet-18 (AP +CF)	Predict MVI in HCC patients	CT	309 patients, Training:216, Validation: 93, External testing: 164	Acc=0.72, Sen=0.96, Spec=0.62, AUC=0.85	Acc=0.71, Sen=0.82, Spec=0.67, AUC=0.78
(72)	2021	SVM (CF)	Predict MVI in HCC patients	CT	309 patients, Training:216, Validation: 93, External testing: 164	Acc=0.77, Sen=0.71, Spec=0.8, AUC=0.78	Acc=0.7, Sen=0.77, Spec=0.67, AUC=0.76
(72)	2021	SVM (AP + CF)	Predict MVI in HCC patients	CT	309 patients, Training:216, Validation: 93, External testing: 164	Acc=0.6, Sen=0.93, Spec=0.46, AUC=0.7	Acc=0.57, Sen=0.9, Spec=0.47, AUC=0.68
(81)	2021	3D-CNN	Predict MVI in HCC patients	CT	405 patients, Training:324, Validation:81	Acc=0.852, Sen=0.932, Spec=0.757, AUC=0.906, F1 score=0.872	~
(135)	2019	ML	Predict HCC recurrence postresection	CECT	470 patients, Training:210, Internal testing: 107; External testing: 153	Pre: AUC=0.84, Post: AUC=0.859	Pre: AUC=0.803, Post: AUC=0.813
(136)	2020	ML	Predict pathological grade of HCC	CECT	297 patients, training:237, test:60	Acc=0.5333, Sen=0.6522, Spec=0.4595, AUC=0.6698	~
(137)	2021	CDLM	Predict MVI in HCC patients	CECT	306 patients, validation:115	Acc=0.73, Sen=0.574, Spec=0.869, AUC=0.736	~
(132)	2022	DL based clinical-radiological model	Predict MVI in HCC patients	CECT	283 patients, Training:198, Testing: 85	Acc=0.9647, Sen=0.9091, Spec=0.9730, Prec=0.894, F1 score=0.870, AUC=0.909	~
(132)	2022	Xception	Predict MVI in HCC patients	CECT	283 patients, Training:198, Testing: 85	Acc=0.7059, Sen=0.6364, Spec=0.7162, Prec=0.432, F1 score=0.359, AUC=0.759	~
(132)	2022	VGG16	Predict MVI in HCC patients	CECT	283 patients, Training:198, Testing: 85	Acc=0.7294, Sen=0.5455, Spec=0.7568, Prec=0.524, F1 score=0.343, AUC=0.639	~
(132)	2022	VGG19	Predict MVI in HCC patients	CECT	283 patients, Training:198, Testing: 85	Acc=0.6824, Sen=0.5455, Spec=0.7027, Prec=0.460, F1 score=0.308, AUC=0.705	~
(132)	2022	ResNet50	Predict MVI in HCC patients	CECT	283 patients, Training:198, Testing: 85	Acc=0.8118, Sen=0.7273, Spec=0.8243, Prec=0.565, F1 score=0.5, AUC=0.880	~
(132)	2022	InceptionV3	Predict MVI in HCC patients	CECT	283 patients, Training:198, Testing: 85	Acc=0.7529, Sen=0.8182, Spec=0.7432, Prec=0.289, F1 score=0.462, AUC=0.724	~
(132)	2022	InceptionResNetV2	Predict MVI in HCC patients	CECT	283 patients, Training:198, Testing:85	Acc=0.7294, Sen=0.5455, Spec=0.7568, Prec=0.339, F1 score=0.343, AUC=0.717	~
(138)	2023	DL-based multi-input CNN	Predict recurrence risk for recurrence-free survival in HCC patients	Muti-phase CT	218 patients, Training:152, Internal validation:66, External validation:74	C-index=0.627	C-index=0.630

AP, arterial phase; CF, clinical factors; C-index, concordance index; cML, conventional machine learning; Multi-modal DNN, multi-modal deep neural network; MVI, microvascular invasion; nnU-Net, 3D neural network; OS, overall survival; TACE, trans-arterial chemoembolization.

**Table 5:** AI-based MRI models for liver and liver tumors segmentation.

Ref	Year	AI Model	Task	Imaging method	Dataset (training/test)	Results
(141)	2012	Iterative watershed algorithm and ANN	Liver segmentation	MRI	115 images	Average Acc=0.94
(142)	2016	3D fast marching algorithm and neural network	Liver tumor segmentation	T1-weighted MRI	Medic Medical Center (10 patients), TCIA (6 patients)	mean volumetric overlap error=0.2743, mean percentage volume error=0.1573, Average surface distance (mm)=0.58, RMS surface distance (mm)=1.20, Maximal surface distance (mm)=6.29
(143)	2018	FCNN	Liver axial segmentation	Late-Phase MRI	Total: 90 patients, Training: 57, Validation: 5, Testing: 20	Dice=0.946 ± 0.018, RVE(%)=4.20 ± 3.34
(143)	2018	FCNN	Liver OrthoMean segmentation	Late-Phase MRI	Total: 90 patients, Training: 57, Validation:5, Testing: 20	Dice= 0.951 ± 0.018, RVE(%)=4.20 ± 3.65
(143)	2018	FCNN	Tumor axial segmentation	Late-Phase MRI	Total: 90 patients, Training set: 57, Validation set: 5, Testing: 20	Dice=0.627 ± 0.241, RVE(%)=48.9 ± 53.3
(143)	2018	FCNN	Tumor OrthoMean segmentation	Late-Phase MRI	Total: 90 patients, Training: 57, Validation: 5, Testing: 20	Dice=0.647 ± 0.210, RVE(%)=35.9 ± 28.2
(144)	2019	2D U-net CNN	Liver segmentation	T1-weighted MRI	498 patients	Dice=0.95 ± 0.03
(144)	2019	2D U-net CNN	Liver segmentation	T2-weighted MRI	498 patients	Dice=0.92 ± 0.05
(145)	2020	Radiomics-guided DUN-GAN	Liver lesion segmentation	multi-phase non-contrast MRI	250 patients	Dice=0.9347
(146)	2020	4D k-means clustering estimation	Liver segmentation	multi-phase MRI	Total: 25 datasets, Training: 10, Validation:15	HH=1.76mm, Dice=0.95, Volume Error =3.18%
(147)	2020	Wide U-Net CNN	Liver Segmentation	T2-weighted MRI	Total: 31 patients	average Dice =0.86 (Liver Vasculature)
(140)	2021	EIS-Net	Liver segmentation	T1-weighted MRI	219 patients, Training:127 Validation: 28 Testing: 44	for tumors <3cm DSC: p = 0.090, MHD: p = 0.385, MAD: p = 0.142
(140)	2021	AS-Net	Liver segmentation	T1-weighted MRI	219 patients, Training:127 Validation: 28 Testing: 44	for tumors >3cm DSC: p = 0.002, MHD: p = 0.003, MAD: p = 0.018
(148)	2021	DCNN+TR+RF	Liver segmentation	T1-weighted MRI	LI-RADS	Validation: Dice=0.91, VOE=17, RVD=-0.04, ASSD (mm)=2.47, MSSD (mm)=25.91, External validation: Dice=0.91, VOE=16, RVD=-0.01, ASSD (mm)=2.67, MSSD (mm)=26.96
(149)	2021	U-net	Segmentation	T2-weighted MRI	Total: 713 patients, Training: 505, Validation:104, Testing:104	Validation: Dice=0.984, Test: Dice=0.983
(150)	2021	United adversarial learning	Liver tumor segmentation and detection	multi-modality NCMRI (T1FS pre-contrast MRI, T2FS MRI, and DWI)	255 subjects	Dice=0.8363, p-Acc=0.9775, IoU=0.813, TPR=0.9213, TNR=0.9375, Acc=0.9294
(150)	2021	Mask R-CNN	Liver tumor segmentation and detection	multi-modality NCMRI (T1FS pre-contrast MRI, T2FS MRI, and DWI)	255 subjects	Dice=0.7517, p-Acc=0.9621, IoU=0.6830, TPR=0.80, TNR=0.832, Acc=0.8157
(150)	2021	FT-MTL-Net	Liver tumor segmentation and detection	multi-modality NCMRI (T1FS pre-contrast MRI, T2FS MRI, and DWI)	255 subjects	Dice=0.7758, p-Acc=0.9648, IoU=0.7064, TPR=0.814, TNR=0.8413, Acc=0.8275
(150)	2021	Tripartite-GAN	Liver tumor segmentation and detection	multi-modality NCMRI (T1FS pre-contrast MRI, T2FS MRI, and DWI)	255 subjects	IoU=0.7342, TPR=0.8682, TNR=0.8968, Acc=0.8824
(150)	2021	Faster R-CNN	Liver tumor segmentation and detection	multi-modality NCMRI (T1FS pre-contrast MRI, T2FS MRI, and DWI)	255 subjects	IoU=0.6643, TPR=0.7863, TNR=0.8226, Acc=0.8039
(150)	2021	U-net	Liver tumor segmentation and detection	multi-modality NCMRI (T1FS pre-contrast MRI, T2FS MRI, and DWI)	255 subjects	Dice=0.7888, p-Acc=0.9657, IoU=0.5833
(150)	2021	Rg-GAN	Liver tumor segmentation and detection	multi-modality NCMRI (T1FS pre-contrast MRI, T2FS MRI, and DWI)	255 subjects	Dice=0.8065, p-Acc=0.9672, IoU=0.6017
(151)	2022	4D DL based on 3D CNN and LSTM	HCC lesion segmentation	T1-weighted MRI	Total: 190 patients, Training: 110, Validation: 40, Internal testing:40	Internal test: Dice=0.825, HD=12.84, VS=0.891, External test: Dice=0.786, HD=21.14, VS=0.89
(151)	2022	3D U-net	HCC lesion segmentation	T1-weighted MRI	Total: 190 patients, Training: 110, Validation: 40, Internal testing:40	Internal test: Dice=0.669, HD=22.39, VS=0.751, External test: Dice=0.604, HD=44.47, VS=0.786
(151)	2022	nnU-net	HCC lesion segmentation	T1-weighted MRI	Total: 190 patients, Training: 110, Validation: 40, Internal testing:40	Internal test: Dice=0.833, HD=10.75, VS=0.88, External test: Dice=0.783, HD=38.61, VS=0.854
(151)	2022	RA-Unet	HCC lesion segmentation	T1-weighted MRI	Total: 190 patients, Training: 110, Validation: 40, Internal testing:40	Internal test: Dice=0.797, HD=23.88, VS=0.87, External test: Dice=0.749, HD=55.60, VS=0.854
(152)	2022	3D CNN	Liver segment segmentation	MRI	Total: 782 patients, Training:367, Validation:157, Testing: 158, Clinical evaluation set: 100	Average Dice=0.902, Average MSD (mm)=3.34, Average HD (mm) =3.61, Average RV= 1.01
(153)	2022	nnU-Net	Lliver parenchyma, portal veins, and hepatic veins segmentation	T1-weighted MRI	30 patients	liver parenchyma: Mean Dice=0.936, portal veins: Median Dice=0.659, hepatic veins: Median Dice=0.548
(139)	2023	Cascaded Network	Liver segmentation	T1-Weighted MRI	CHAOS	Dice=0.9515, IoU=0.921, Acc=0.997
(139)	2023	Deep action learning with 3D UNet	Liver segmentation	T1-Weighted MRI	CHAOS	Dice=0.806
(139)	2023	Contrastive Semi Supervised Learning Approach with UNet	Liver segmentation	T1-Weighted MRI	CHAOS	Dice=0.859
(139)	2023	W-Net with attention gates	Liver segmentation	T1-Weighted MRI	CHAOS	Dice=0.8812
(139)	2023	Source Free Unsupervised UNet	Liver segmentation	T1-Weighted MRI	CHAOS	Dice=0.8840
(139)	2023	Bidirectional Searching Neural Net	Liver segmentation	T1-Weighted MRI	CHAOS	Dice=0.898
(139)	2023	Mask R-CNN	Liver segmentation	T1-Weighted MRI	CHAOS	Dice=0.8
(139)	2023	Geomatric Edge Enhancement based Mask R-CNN	Liver segmentation	T1-Weighted MRI	CHAOS	Dice=0.91
(154)	2023	UNet + +	Liver segmentation	MRI	Total: 105 patients Training set: 83, Validation set: 11, Internal testing:11	Validation: average Dice=0.91, Internal testing: average Dice=0.92
(154)	2023	UNet + +	Liver tumor segmentation	MRI	Total: 105 patients Training: 83, Validation: 11, Internal testing:11	Validation: average Dice=0.612, Internal testing: average Dice=0.687
(155)	2023	nnU-Net	Liver and liver vessles segmentation	T1-weighted MRI	Total: 170 patients Training set: 136, Validation set:34	Dice=0.77, ASSD=3.235, HD95 = 11.276
(156)	2024	3D residual U-Net	Liver segmentation	MRCP	250 (225/25)	Dice=0.8
(140)	2024	DCNN	Liver segmentation	T1-weighted MRI	470 patients, Training set: 329, Validation set: 70, Internal testing: 71 External validation set: LiverHccSeg dataset	Training: mean Dice=0.968, mean MHD=1.876, mean MAD=0.538 Validation: mean Dice=0.966, mean MHD=1.949, mean MAD=0.541 Internal testing: mean Dice=0.967, mean MHD=1.852, mean MAD=0.545 External testing: mean Dice=0.962, mean MHD=2.711, mean MAD=0.705 Public testing: mean Dice=0.928, mean MHD=6.893, mean MAD=1.625
(157)	2024	Isensee 2017 network	Liver segmentation	T1-weighted MRI, T2-weighted MRI	128 patients	average Dice =0.88
(157)	2024	Isensee 2017 network	Liver tumor segmentation	T1-weighted MRI, T2-weighted MRI	128 patients	average Dice =0.53

ANN, artificial neural network; AS-Net, all-stage-net; ASSD, average symmetric surface distance; CHAOS, combined healthy abdominal organ segmentation grant challenge; EIS-Net, early-intermediate-stage-net; HD95, Hausdorff Distance 95; MBH T2WI, conventional multi-breath-hold (MBH) T2WI; MICCAI, medical image computing and computer assisted intervention; NCMRI, multi-modality non-contrast magnetic resonance imaging; Radiomics-guided DUN-GAN, radiomics-guided densely-UNet-nested generative adversarial networks; SBH-T2WI, single-breath-hold T2-weighted MRI; TCIA, the cancer imaging archive.

**Table 6:** AI-based MRI models for diagnosing HCC.

Ref	Year	AI Model	Tasks	Imaging method	Dataset	Internal Testing Results	External Testing Results
(160)	2019	3D CNN	Discriminating primary and metastatic liver tumors	diffusion weighted MRI (DW-MRI)	Training: 74, Validation: 33, Testing: 23	Acc=0.83, Average Prec=0.75, AUC=0.80, Spec=0.67, Sen=0.93, Prec=0.83, Fe-score=0.83	~
(159)	2019	CNN	Classify liver lesions (six types)	multi-phasic MRI	Training:434, Testing:60	Acc=0.897, Prec=0.722, Recall=0.826	~
(158)	2019	CNN-based DLS	Classify FLLs include HCC	multi-phasic MRI	Training:434, Testing:60	Overall Acc=0.90, Overall Sen=0.94, Overall Spec=0.97	~
(158)	2019	CNN-based DLS	Classify common hepatic lesions	T1-weighted MRI	Training:434, Testing:60	Acc=0.943	Acc=0.92, Sen=0.92, Spec=0.98
(165)	2019	Extremely randomized trees classifier	Classify FLLs (five types)	T2-weighted MRI	95 patients	Overall Acc=0.77	~
(16)	2020	AlexNet+ transfer learning	distinguish LI-RADS grade 3 liver tumors from combined higher-grades 4 and 5 tumors for HCC diagnosis	multiphase MRI	LI-RADS dataset, Training (60%), Validation (20%), Testing (20%)	Acc=0.90, Sen=1.0, Prec=0.835, AUC=0.95	~
(161)	2020	CNN	Classify HCC	MRI	Total: 1210 patients (31608 images), External validation: 201 patients (6816 images)	AUC=0.951, Sen=0.919, Spec=0.941	~
(161)	2020	CNN+ clinical data	Classify HCC	MRI	Total: 1210 patients (31608 images), External validation: 201 patients (6816 images)	AUC=0.951, Sen=0.957, Spec=0.904	~
(161)	2020	CNN+ clinical data	Classify metastatic malignancy	MRI	Total: 1210 patients (31608 images), External validation: 201 patients (6816 images)	AUC=0.985, Sen=0.946, Spec=1	~
(161)	2020	CNN+ clinical data	Classify primary malignancy except HCC	MRI	Total: 1210 patients (31608 images), External validation: 201 patients (6816 images)	AUC=0.905, Sen=0.733, Spec=0.964	~
(148)	2021	DCNN	Detect HCC	T1-weighted MRI	LI-RADS	Sen_20 = 0.73, Sen_50 = 0.55, AFPR=2.81, Dice=0.4	~
(148)	2021	DCNN+TR	Detect HCC	T1-weighted MRI	LI-RADS	Sen_20 = 0.73, Sen_50 = 0.55, AFPR=0.77, Dice=0.49	~
(148)	2021	DCNN+RF	Detect HCC	T1-weighted MRI	LI-RADS	Sen_20 = 0.73, Sen_50 = 0.55, AFPR=0.85, Dice=0.47	~
(148)	2021	DCNN+TR+RF	Detect HCC	T1-weighted MRI	LI-RADS	Sen_20 = 0.73, Sen_50 = 0.55, AFPR=0.62, Dice=0.49	Sen_20 = 0.75, Sen_50 = 0.66, AFPR=0.75, Dice=0.48
(149)	2021	ResNet50	Liver cirrhosis identification	T2-weighted MRI	Total: 713 patients, Training: 505, Validation:104, Testing:104	Acc=0.99, Sen=0.98, Spec=0.96	Acc=0.96, Sen=0.98, Spec=0.79
(149)	2021	DTL	Liver cirrhosis classification	T2-weighted MRI	Total: 713 patients, Training: 505, Validation:104, Test:104	Acc=0.88	Acc=0.91
(166)	2020	CNN	Detect HCC	MRI	Training:455 patients, Testing:45 patients	Sen=0.87, Spec=0.93, AUC=0.90	~
(166)	2020	CNN	Classify FLLs	MRI	Training:1210 patients, Testing:201 patients	Sen=0.405~1, Spec=0.673~1, AUC=0.841−0.989	~
(166)	2020	CNN	Distinction LI-RADS 3 & LI-RADS 4/5 tumors	MRI	89 images from 59 patients	Acc=0.767~0.9, Sen=0.756~0.889	~
(166)	2020	CNN	Classify HCC & non-HCC lesions	MRI	Training:140 patients, Testing:10 patients	Acc=0.873, Sen=0.82, Spec=0.927	~
(166)	2020	CNN RF	HCC detection	MRI	171 patients	Dice=0.48, Sen=0.66~0.75	~
(167)	2021	GoogLeNet (Inception-V1)	Classify HCC & normal histopathology images	MRI	29 patients	Acc=0.9137, Sen=0.9216, Spec=0.9057	~
(164)	2021	CNN	Classify HCC	MRI	118 patients	Overall Acc=0.873	~
(164)	2021	CNN	Classify non-HCC	MRI	118 patients	Acc=0.941, Sen=0.82, Spec=0.927	~

AFP, α-fetoprotein; AFPR, the average false positive rate; CDLM, combined deep learning model; cMRI, conventional magnetic resonance imaging (including T2 + DWI + DCE); DCE, dynamic contrast enhanced; DLCR, deep learning combined radiomics; DLF, deep learning features; DTL, deep transfer learning; DW-MRI, diffusion weighted MRI; EOB-MRI, gadoxetic acid-enhanced magnetic resonance imaging; i-RAPIT, intelligent-augmented model for risk assessment of post liver transplantation; LASSO, the least absolute shrinkage and selection operator; LI-RADS, liver imaging reporting and data system; MCAT, multimodality-contribution-aware TripNet; MRE, magnetic resonance elastography; PD-L1, programmed death-ligand 1.

**Table 7:** AI-based MRI models for HCC prognostication.

Ref	Year	AI Model	Tasks	Imaging modality	Dataset	Internal Testing Results	External Testing Results
(170)	2021	First CapsNet Network	Predict survival outcomes on liver transplantation patients with HCC	MRI	Training:87 patients, Testing:22 patients	Acc=0.64, F1 score=0.61	~
(168)	2021	H-DARnet	Predict MVI in HCC patients	T2-weighted MRI	Training:168 patients, Testing:57 patients	Acc=0.826, Sen=0.795, Spec=0.738, AUC=0.775	~
(168)	2021	Vgg19	Predict MVI in HCC patients	T2-weighted MRI	Training:168 patients, Testing:57 patients	Acc=0.505, Sen=0.446, Spec=0.629, AUC=0.537	~
(168)	2021	AlexNet	Predict MVI in HCC patients	T2-weighted MRI	Training:168 patients, Testing:57 patients	Acc=0.515, Sen=0.446, Spec=0.662, AUC=0.573	~
(168)	2021	SqueezeNet	Predict MVI in HCC patients	T2-weighted MRI	Training:168 patients, Testing:57 patients	Acc=0.54, Sen=0.461, Spec=0.708, AUC=0.625	~
(168)	2021	ResNet50	Predict MVI in HCC patients	T2-weighted MRI	Training:168 patients, Testing:57 patients	Acc=0.545, Sen=0.453, Spec=0.746, AUC=0.626	~
(168)	2021	GoogleNet	Predict MVI in HCC patients	T2-weighted MRI	Training:168 patients, Testing:57 patients	Acc=0.605, Sen=0.553, Spec=0.713, AUC=0.649	~
(168)	2021	DenseNet121	Predict MVI in HCC patients	T2-weighted MRI	Training:168 patients, Testing:57 patients	Acc=0.625, Sen=0.586, Spec=0.711, AUC=0.678	~
(168)	2021	SE-DenseNet121	Predict MVI in HCC patients	T2-weighted MRI	Training:168 patients, Testing:57 patients	Acc=0.705, Sen=0.753, Spec=0.60, AUC=0.738	~
(168)	2021	Simple-SE-DenseNet	Predict MVI in HCC patients	T2-weighted MRI	Training:168 patients, Testing:57 patients	Acc=0.735, Sen=0.754, Spec=0.696, AUC=0.769	~
(187)	2021	Fusion DL model	Predict MVI in HCC patients	EOB-MRI	Training:329 patients; external test: 115 patients	~	Acc=0.757, Sen=0.704, Spec=0.803, AUC=0.802
(187)	2021	CDLM	Predict MVI in HCC patients	EOB-MRI	Training:329 patients; external test: 115 patients	~	Acc=0.757, Sen=0.704, Spec=0.803, AUC=0.812
(171)	2021	DLF	Predict PD-L1 expression level in HCC patients	T2-weighted MRI	103 patients	5-Fold cross validation: Acc=0.854, F1-score=0.703, Spec=0.947, Prec=0.892, Recall=0.633, AUC=0.852	~
(171)	2021	radiomics-based model+DLF	Predict PD-L1 expression level in HCC patients	T2-weighted MRI	103 patients	5-Fold cross validation: Acc=0.887, F1-score=0.764, Spec=0.981, Prec=0.948, Recall=0.660, AUC=0.897	~
(172)	2023	SVM	Predict MVI in HCC patients	multi-parameter MRI	Training:297 patients, Testing: 100 patients	Acc=0.64, Sen=0.8065, Spec=0.5652, AUC=0.766	~
(172)	2023	ResNet18	Predict MVI in HCC patients	multi-parameter MRI	Training:297 patients, Testing: 100 patients	Acc=0.73, Sen=0.7097, Spec=0.7391, AUC=0.7938	~
(169)	2023	KNN	Predict TACE outcomes for HCC patients	T2-weighted MRI	Training: 115 patients, Testing; 29 patients	Acc=0.655, Sen=0.538, Spec=0.75, AUC=0.669	Acc=0.536, Sen=0.857, Spec=0.357, AUC=0.615
(169)	2023	SVM	Predict TACE outcomes for HCC patients	T2-weighted MRI	Training: 115 patients, Testing; 29 patients	Acc=0.621, Sen=0.769, Spec=0.563, AUC=0.688	Acc=0.679, Sen=0.786, Spec=0.714, AUC=0.712
(169)	2023	Lasso	Predict TACE outcomes for HCC patients	T2-weighted MRI	Training: 115 patients, Testing; 29 patients	Acc=0.655, Sen=0.769, Spec=0.813, AUC=0.745	Acc=0.679, Sen=0.929, Spec=0.5, AUC=0.663
(169)	2023	DNN	Predict TACE outcomes for HCC patients	T2-weighted MRI	Training: 115 patients, Testing; 29 patients	Acc=0.759, Sen=0.923, Spec=0.688, AUC=0.837	Acc=0.714, Sen=0.714, Spec=0.857, AUC=0.796

CDLM, contrast-dependent learning model; EOB-MRI, gadoxetic acid-enhanced MRI; MVI, Microvascular Invasion; TACE, transarterial chemoembolization.

**Table 8:** Summary of studies evaluating AI-based multi-modal models for liver and liver tumors segmentation.

Ref	Year	AI Model	Task	Imaging method	Dataset	Results
(144)	2019	2D U-Net	Liver segmentation	T1-weighted MRI+ T2-weighted MRI+CT	Total: 498 subjects	CT: Dice=0.94 ± 0.06, T1-weighted MRI: Dice=0.95 ± 0.03, T2-weighted MRI: Dice=0.92 ± 0.05
(174)	2019	CycleGAN- DADR	Liver segmentation	CT+MRI	LiTS+multi-phasic MRI images of 20 patients with HCC	Dice=0.74
(112)	2021	APA2Seg-Net	Liver segmentation	CBCT+MRI	LiTS	CBCT: Median Dice=0.903, Mean Dice=0.893, Median ASD=5.882, Mean ASD=5.886, MRI: Median Dice=0.918, Mean Dice=0.921, Median ASD=1.491, Mean ASD=1.860
(175)	2022	Unsupervised domain adaptation framework	Liver segmentation	MRI+CT	LiTS+ CHAOS	Dice= 0.912 ± 0.037
(173)	2023	SWTR-Unet	Joint liver and hepatic lesion segmentation	MRI+CT	61440 MRI images + 189600 CT images	Dice_liver_=0.98 ± 0.02, Dice_lesion_=0.81 ± 0.28, HD_liver_=1.02 ± 0.18, HD_lesion_=7.03 ± 17.37

CycleGAN- DADR, CycleGAN based domain adaptation via disentangled representations.

**Table 9:** AI-based multi-modal models for diagnosing HCC.

Ref	Year	AI Model	Task	Imaging modality	Dataset	Results
(176)	2020	DCNN	Diagnosis HCC	CT + 20 biological markers	Total: 766 Training:536 Validation: 153 Testing:77	
(161)	2020	Google Inception-ResNet V2 CNN + autoencoder neural network CNN	Diagnosis HCC	MRI + 16 biological markers	Total: 38424 images Training:31608 images from 1210 patients Validation: 6816 images from 201 patients	AUC=0.946 for distinguishing malignant from benign liver tumors, AUC=0.985 for classifying HCC AUC=0.998 for classifying metastatic tumors, AUC=0.963 for classifying other primary malignancies
(177)	2021	Xception CNN	Diagnosis HCC	CT+ 20 clinical parameters	Total: 37084 Training: 29104, Validation: 3816, Testing:4164	Acc = 0.869, Prec =0.896, Recall =0.869, F1 score =0.867
(118)	2021	STIC	Classify HCC and ICC	Multi-phase CECT+clinical data	Total: 723 patients, Training:499, Testing:113, External testing: 111	Acc=0.862, AUC=0.893
(178)	2021	STIC	Diferential diagnosis of malignant hepatic tumors	Multi-phase CECT+clinical data	Total: 723 patients, Training:499, Testing:113, External testing: 111	Acc=0.726,
(179)	2021	SVM	Classify aHCC and FNH	CEUS+ radiologist’s	266 patients	AUC=0.93, Sen=0.935, Spec=0.849
(180)	2022	DL	Classify benign and malignant liver lesions	CEUS+clinical factors	303 patients	AUC=0.957, Acc=0.94, Sen=0.966, Spec=0.905
(181)	2023	Multi-modal DNN + Transfer learning & fine-tuned	Multi-class liver cancer diagnosis	CT+ pathology data		Average Acc=0.9606, AUC=0.832

**Table 10:** AI-based multi-modal models for prognostication of HCC.

Ref	Year	AI Model	Task	Imaging method	Dataset	Results
(182)	2020	Cox-PH	Predict MVI in HCC patients	CT + 9 clinical parameters	Total:145 Training set: 145	AUC=0.79
(183)	2021	GhostNet/CNN	Predict TACE response for HCC therapy	CT + clinical evaluation (clinical parameters and biological markers)	Training:319 patients, Validation: 80 patients	AUC=0.98, Acc=0.98
(170)	2021	First CapsNet network + Second CapsNet network	Predict survival outcomes on liver transplantation patients with HCC	MRI + pathology	Training:87 patients, Testing:22 patients	Acc=0.68, F1 score=0.65
(170)	2021	First CapsNet network + RBF network	Predict survival outcomes on liver transplantation patients with HCC	MRI + Clinical signatures	Training:87 patients, Testing:22 patients	Acc=0.78, F1 score=0.75
(170)	2021	Second CapsNet network + RBF network	Predict survival outcomes on liver transplantation patients with HCC	Pathology + clinical	Training:87 patients, Testing:22 patients	Acc=0.77, F1 score=0.73
(170)	2021	i-RAPIT	Predict survival outcomes on liver transplantation patients with HCC	Clinical+MRI+pathology features	Training:87 patients, Testing:22 patients	Acc=0.87, F1 score=0.84, Recall=0.80, Prec=0.89
(184)	2021	Radiomics, CNN	Predict MVI in HCC patients	MRI + 22 clinical parameters	Total: 601 Training set:461 Test set:140	AUC= 0.915, Overall Acc=0.793
(133)	2021	UNet, radiomics, multi-task deep learning neural network (MTNet)	Predict MVI in HCC patients	CT+ 22 biological markers	Total: 366 Training set:281 Validation set: 85	Training set: AUC=0.877, Validation set: AUC=0.836
(185)	2022	Baseline+MCAT	Histologic grading of HCC	T2-weighted MRI + T1-weighted MRI +DCE MRI	59 patients	Acc=0.8344, Sen=0.8725, Prec=0.8942, F1-score=0.8877
(185)	2022	Baseline+MAWM	Histologic grading of HCC	T2-weighted MRI + T1-weighted MRI +DCE MRI	59 patients	Acc=0.7922, Sen=0.8291, Prec=0.8197, F1-score=0.8382
(185)	2022	Baseline+TripNet	Histologic grading of HCC	T2-weighted MRI + T1-weighted MRI +DCE MRI	59 patients	Acc=0.7854, Sen=0.7944, Prec=0.8235, F1-score=0.7867
(186)	2022	DLCR	Predict Ki-67 expression in HCC patients	cMRI + AFP	Total: 108 patients, Training: 87 patients, Internal validation:21 patients External Testing: 43 patients	Validation: Acc=0.81, Sen=0.80, Spec=0.82, PPV=0.78, NPV=0.80, AUC=0.84 External Testing: Acc=0.72, Sen=0.72, Spec=0.72, PPV=0.68, NPV=0.71, AUC=0.74
(186)	2022	DLCR	Predict Ki-67 expression in HCC patients	cMRI + AFP + MRE	Total: 108 patients, Training: 87 patients, Internal validation:21 patients external Testing: 43 patients	Validation: Acc=0.87, Sen=0.86, Spec=0.93, PPV=0.84, NPV=0.87, AUC=0.90 External Testing: Acc=0.83, Sen=0.80, Spec=0.86, PPV=0.78, NPV=0.80, AUC=0.83
(138)	2023	ResNet18	Predict MVI in HCC patients	CT+multi-parameter MRI	Training:297 patients, Testing: 100 patients	Traing: Acc=0.8923,Sen=0.8908, Spec=0.8933, AUC=0.9558, Testing: Acc=0.8, Sen=0.7742, Spec=0.8116, AUC=0.8191
(138)	2023	ResNet18 +SVM	Predict MVI in HCC patients	CT+multi-parameter MRI	Training:297 patients, Testing: 100 patients	Traing: Acc=0.9293, Sen=0.9160, Spec=0.9382, AUC=0.9804 Testing: Acc=0.82, Sen=0.7742, Spec=0.8406, AUC=0.8415

APA2Seg-Net, anatomy-preserving domain adaptation to segmentation network; Cox-PH, Cox-proportional hazard; STIC, spatial extractor-temporal encoder-integration-classifier; SWTR-Unet, SWIN-transformer-Unet.

## 3 Artificial intelligence techniques

AI techniques, including Machine Learning (ML) and Deep Learning (DL), have been extensively investigated in application and interest within the field of liver cancer research (187–190). ML utilizes data to develop algorithms that can identify specific behavioral patterns and build predictive models. The objective of ML is to create a model that leverages statistical dependencies and correlations within a dataset, eliminating the need for explicit programming. This process is divided into two stages: training and validation. During the training stage, the model is exposed to a portion of the available data (training dataset). In the validation stage, the model’s performance is evaluated on a separate subset of the dataset (test dataset) to assess its ability to generalize its training performance to unseen data. Well-known ML algorithms, such as Support Vector Machines (SVM) and Artificial Neural Networks (ANNs), have been applied in HCC management (191, 192).

DL technology, a subset of ML, has shown remarkable efficacy in the analysis of liver images. This is largely due to its ability to process large volumes of data through multiple layers of artificial neurons. These neurons are engineered to emulate the intricate structure of the human brain and its biological neural networks. A unique characteristic of DL algorithms is that these layers of features are not manually constructed with human expertise. Rather, they are autonomously learned from data using a general-purpose learning procedure. This facilitates an end-to-end mapping from the input to the output, essentially converting the image into classification methods. In ML methods, success is contingent upon accurate segmentation and the selection of expert-designed features. DL approaches can surmount these limitations as they can identify the regions of the image most associated with the outcome through self-training. Moreover, they can discern the features of the region that informed the decision through multiple layers.

Convolutional Neural Networks (CNNs) are presently the most prevalent DL algorithms employed for the diagnosis and management of HCC (193–195). The uniqueness of CNNs compared to Fully Connected Networks lies in their ability to capture spatial hierarchies through convolutional and pooling layers, their parameter efficiency due to shared weights, and their effectiveness in processing structured data like images and videos. The fundamental principles of CNNs include local connections, shared weights, pooling, and the use of numerous layers. These components collectively enhance the accuracy and efficiency of the entire system. A standard CNN model is composed of an input layer, an output layer, and several hidden layers. These hidden layers encompass convolutional layers, pooling layers, and fully-connected layers. By repeatedly applying convolution and pooling, fully-connected layers are subsequently utilized for classification or predictions. There exists a variety of layer combinations, and numerous Deep Neural Network (DNN) architectures have been successfully implemented for HCC diagnosis and prediction. These include Fully Convolutional Networks (FCNs) (196), 3D U-Net (197), Recurrent Neural Networks (RNNs) (198), Graph Convolutional Networks (GCNs) (199, 200), Generative Adversarial Networks (GANs) (16, 201), AlexNet (202), and VGGNet-19 (203). These models are specifically engineered to eliminate fully connected layers and restore spatial dimensions, thereby augmenting DL capabilities even when there is a scarcity of labeled data. However, it is imperative to address domain adaptation and dataset bias to ensure the success of transfer learning (TL). This is because these factors can significantly influence the performance and generalizability of the models.

In contrast to CNNs, Fully Convolutional Networks (FCNs) are engineered to preserve spatial information, thereby enhancing their effectiveness for pixel-level predictions. This attribute renders FCNs particularly apt for liver tumor segmentation, as they employ convolutional layers in lieu of fully connected ones (196).

U-Net, conversely, utilizes an encoder-decoder model equipped with skip connections. This architecture enables it to amalgamate local and global context information, thereby augmenting object localization precision. Despite the limitations posed by scarce training data, 3D U-Net has exhibited remarkable results in the classification of liver lesions (197).

RNNs, encompassing Long Short-Term Memory (LSTM) and Gated Recurrence Unit (GRU), are specifically tailored to scrutinize sequential data by capturing temporal dependencies. These models have been successfully deployed for predicting HCC recurrence post liver transplantation (198). By addressing the vanishing gradients issue and capitalizing on temporal dependencies, they have substantially enhanced prediction accuracy.

Graph Convolutional Networks (GCNs) offer a variety of techniques for graph convolution, which are instrumental in clinically predicting Microvascular Invasion (MVI) in Hepatocellular Carcinoma (HCC) (199). These techniques include spectral-based and spatial-based GCN approaches, each carrying unique computational implications. DenseGCN, a contemporary architecture, has been introduced for the identification of liver cancer. It integrates advanced techniques such as similarity network fusion and denoising autoencoders, significantly boosting detection accuracy (200).

Generative Adversarial Networks (GANs) have demonstrated their value in generating synthetic images and augmenting data across a range of medical applications. In the realm of liver tumor detection, Tripartite GAN offers a cost-effective and non-invasive alternative by generating contrast-enhanced MRI images, eliminating the need for contrast agent injection (201). Another promising application is the Mask-Attention GAN, which generates realistic tumor images in CT scans for training and evaluation purposes (16).

Transfer Learning (TL) strategies have been employed in the field of medical imaging to mitigate overfitting issues arising from limited data. Within the TL framework, knowledge can be shared and transferred between different tasks. The workflow comprises two steps: pretraining on a large dataset and fine-tuning on the target dataset. Essentially, by fine-tuning the DL architecture, the knowledge gleaned from one dataset can be transferred to a dataset procured from another center.

## 4 AI-based US techniques

US is recommended in clinical guidelines for the detection of HCC in patients with cirrhosis. However, its efficacy can be influenced by several factors, including operator experience, equipment quality, and patient morphology. Previous studies have indicated that the sensitivity of HCC detection using conventional US ranges from 59% to 78% (204). To enhance sensitivity and specificity, various US modalities have been explored. For instance, Contrast-Enhanced Ultrasound (CEUS) has been demonstrated to improve the sensitivity of HCC detection. These models serve as invaluable tools for predicting HCC recurrence, guiding treatment decisions, and improving patient outcomes. This study investigates the most recently developed AI-based approaches for evaluating detection, prognostication, treatment response, and survival in HCC. **Table 1** provides a summary of the results from studies evaluating AI-based US approaches for HCC diagnosis.

### 4.1 Diagnosis of focal liver lesions

This section outlines the recently developed AI-based US models for diagnosing HCC. These applications encompass diagnosing focal liver lesions (FLLs), distinguishing between benign and malignant liver lesions, differentiating HCC from focal nodular hyperplasia (FNH), cirrhotic parenchyma (PAR), and intrahepatic cholangiocarcinoma (ICC) (see **Table 1**). Among these studies, Bharti et al. (21) proposed a Support Vector Machine (SVM) model that integrates three classifiers using B-mode US data to assess and differentiate various stages of liver disease, achieving a classification accuracy of 96.6%.

In 2020, Brehar et al. (24) demonstrated that a CNN model, trained on two distinct US machine datasets (GE9 and GE7), surpassed conventional ML models (SVM, Random Forest (RF), Multi-Layer Perceptron, and AdaBoost) in differentiating between HCC and PAR. The proposed model achieved Area Under the Curve (AUC) values of 0.91 and 0.95 and accuracies of 84.84% and 91% in the GE9 and GE7 datasets, respectively. In 2023, Jeon et al. (35) proposed a CNN model using quantitative US data from 173 patients for diagnosing hepatic steatosis, achieving an AUC of 0.97, a sensitivity of 90%, and a specificity of 91%.

CEUS generally outperforms B-mode US in diagnosing FLLs and HCC, and AI has augmented its capabilities in identifying potential malignancies. Several research groups have studied the differentiation of benign and malignant FLLs (refer to **Table 1**). In 2020, Huang et al. (43) investigated the use of an SVM model for evaluating diagnostic accuracy when differentiating between atypical HCCs (aHCC) and FNH using CEUS data. The proposed SVM model achieved an AUC of 0.944, a sensitivity of 94.76%, and a specificity of 93.62%.

In 2021, Căleanu et al. (44) proposed a DL model to classify five types of FLLs using CEUS data, obtaining a general accuracy of 88%. Hu et al. (45) investigated a CNN model trained on four-phase CEUS video data from 363 patients. The proposed CNN model achieved an accuracy of 91% and an AUC of 0.934 on the testing dataset, slightly outperforming resident radiologists and matching experts.

### 4.2 Characterization of focal liver lesions

In a study conducted by Virmani et al. (7), a Neural Network Ensemble (NNE) model was proposed to distinguish a normal liver from four distinct liver lesions, achieving an impressive accuracy of 95%. The diagnoses for the included liver lesions were confirmed through experienced radiologists, clinical follow-ups, and other associated findings.

In 2017, Hassan et al. (20) introduced an ANN model that achieved a classification accuracy of 97.2% for benign and malignant FLLs. In 2019, Schmauch et al. (22) developed a supervised DL model, specifically a CNN, utilizing a French radiology public challenge dataset for diagnosing FLLs. The model was capable of detecting FLLs and categorizing them as benign (such as cyst, FNH, and angioma) or malignant (like HCC, metastasis), achieving a mean AUC of 0.935 and 0.916 in the training dataset. Despite promising results, further validation is required due to the limited number of images used for training.

In 2020, Yang et al. (23) conducted a multicenter study to develop a Deep Convolutional Neural Network (DCNN) using an US database, along with background and clinical parameters (such as HBV, HCV, lesion margin, morphology) for characterizing FLLs. The model achieved an AUC of 0.924 for distinguishing benign from malignant lesions in the external validation dataset. The model demonstrated superior accuracy compared to clinical radiologists and CECT, albeit slightly lower than Contrast-Enhanced Magnetic Resonance Imaging (CE-MRI) (87.9%). This approach could potentially enhance radiologists’ performance and reduce the reliance on CECT/CEMR and biopsy.

In 2021, Mao et al. (25) developed various ML-based models for distinguishing primary liver cancer and secondary liver cancer by extracting radiomic features from US images. The Logistic Regression (LR) model outperformed other ML models in this study. Ren et al. (30) applied a Support Vector Machine (SVM) model in B-mode US for predicting the pathological grading of HCC, achieving an AUC of 0.874 in the test set. The same research group also developed another SVM model for differentiating HCC from Intrahepatic Cholangiocarcinoma (ICC), yielding good performances (31). In these studies, liver lesions were pathologically confirmed and used as the standard reference.

In 2017, Guo et al. (40) demonstrated that a multiple-kernel learning-based model could enhance the sensitivity, specificity, and overall accuracy of CEUS for detecting HCC. Later, Ta et al. (41) proposed an ANN model using CEUS data for differentiating benign liver lesions from malignant ones. The model showed promising results, classifying liver lesions as benign or malignant with accuracy comparable to expert radiologists and superior to physicians. Huang et al. (43) constructed an SVM model for differentiating atypical HCC (aHCC) and FNH using CEUS data, achieving an average accuracy of 94.4% compared to pathology reports and clinical follow-up.

In 2021, Wang et al. (46) proposed an SVM model using CEUS data, which could discriminate HCC pathological grading with an AUC of 0.72. More recently, Zhou et al. (48) investigated CNN-Long Short-Term Memory (LSTM), 3D CNN, and ML-TIC models for classifying benign and malignant liver lesions using CEUS data from 440 patients, achieving AUC values of 0.91, 0.88, and 0.78, respectively.

### 4.3 Evaluation prognostication, treatment response and survival in HCC

Surgery, Transcatheter Arterial Chemoembolization (TACE), and Microwave Ablation are widely recognized as treatment methods for liver cancer. Each method requires meticulous candidate evaluation to ensure optimal therapeutic effectiveness (38–40). Wu et al. (203) employed ResNet18 in B-mode US to predict HCC recurrence after Microwave Ablation. The model achieved C-index values of 0.695, 0.715, 0.721, and 0.721 for early relapse, late relapse, and relapse-free survival in HCC patients, respectively.

Liu et al. (42) developed two DL-based models using CEUS data to predict the two-year progression-free survival of HCC patients undergoing either Radiofrequency Ablation or Surgical Resection. The models achieved C-index values of 0.726 and 0.741 for Radiofrequency Ablation and Surgical Resection, respectively. When the Surgical model was applied to predict outcomes for patients initially treated with Ablation, it suggested that approximately 17.3% of Ablation patients could potentially experience a longer two-year progression-free survival if they underwent Surgery. Conversely, the Ablation predictive model indicated that 27.3% of Surgical patients might achieve a longer two-year progression-free survival if they had received Ablation treatment. These CEUS-based models provide accurate survival assessments for HCC patients and facilitate optimal treatment selection. Furthermore, the same research group employed a DL model to quantitatively analyze CEUS videos (43). They developed three models to predict personalized responses of HCC patients after their first TACE session. The CEUS-based model outperformed the other two ML models, achieving a higher AUC value (0.93 vs 0.80 vs 0.81).

In another study, Ma et al. (44) applied a Radiomics model in dynamic CEUS to predict early and late recurrence in patients with an HCC lesion less than 5cm in diameter after Thermal Ablation. The prediction model yielded an AUC of 0.84 for early recurrence and a C-index of 0.77 for late recurrence in the test group. The proposed model, which combines CEUS, US Radiomics, and clinical factors, performed well in predicting early HCC recurrence after Ablation and could stratify the high risk of late recurrence.

Lastly, Liu et al. (16) introduced DL models in CEUS to predict the two-year progression-free survival rate of HCC patients, demonstrating exceptional accuracy in guiding treatment decisions. Other researchers have incorporated additional pattern recognition classifiers into DCNN algorithms using CEUS to improve the diagnosis of FLLs. However, previous studies only involved small sample sizes, thus standardized imaging data or external validations are required to validate the model’s generalizability across populations.

## 5 AI-based CT techniques

Numerous research groups have explored the application of AI in liver cancer research, specifically leveraging CT scan technology. This section delves into AI-based CT methodologies for diagnosing and predicting HCC. **Tables 2** and **3** encapsulate selected studies, which can be categorized into three distinct groups: segmentation of liver and liver tumors, characterization of FLLs, and evaluation of prognostication, treatment response, and survival in HCC patients.

### 5.1 Segmentation of liver and liver tumors

The segmentation of liver and liver tumors plays a crucial role in assessing tumor burden, detecting early recurrence, extracting image features, and formulating treatment plans. The manual segmentation of liver and liver lesions is a significant challenge and is time-consuming due to the extensive range of radiographic features in HCC. AI-driven CT models have emerged as powerful tools for the automatic segmentation of liver and liver tumors. **Table 2** provides a summary of recently developed AI-driven CT models for segmentation of liver and liver tumors.

In 2015, Li et al. (49) introduced a DCNN for the segmentation of liver tumors in CT scans, achieving a precision rate of 82.67%. In 2017, Vivanti et al. (50) examined a CNN-based segmentation model for the automatic detection of recurrence during follow-up, achieving a true positive rate of 86% for lesions larger than 5 mm (28). Subsequently, Sun et al. (51) and Das et al. (52) conducted comprehensive studies on the automatic segmentation of tumors in the liver using CNN-based architectures such as Fully Convolutional Networks (FCNs) and U-Net. In 2017, Sun et al. (51) proposed an FCNs model for the segmentation of liver tumors, achieving high accuracy.

Since 2017, the Liver Tumor Segmentation Challenge (LiTS) has been encouraging researchers to create AI models for the automatic segmentation of liver tumors. This challenge utilizes a multinational dataset of CT images, known as LiTS17, which includes 130 CT images for training and 70 CT images for testing. Over the past few years, this challenge has seen participation from more than 280 research teams worldwide, with models based on Fully Convolutional Networks (FCN) or U-Net achieving top scores for the segmentation of liver and liver tumors.

At present, the highest-scoring model, MAD-UNet (83), has achieved Dice score of 0.9727 for the segmentation of liver using the LiTS17 dataset. While these results are promising, there is a notable variability in both the imaging characteristics of liver tumors and their delineation. This highlights the need for universal and standardized methods for liver tumor segmentation.

### 5.2 Characterization of focal liver lesions

**Table 3** summarizes the results of studies that have evaluated AI-based CT models for diagnosing HCC. Mokrane et al. (106) developed a ML model using 13,920 CT images from 189 patients. This model was able to distinguish HCC from non-HCC lesions in cirrhotic patients, achieving AUC values of 0.81 and 0.66 in the training and external validation datasets, respectively.

In 2019, Khan et al. (107) developed a SVM model that classified FLLs as benign or malignant, achieving an accuracy of 98.3%. Das et al. (52) proposed a CAD system based on a watershed transform and Gaussian Mixture Model (GMM) for accurate and automated liver lesion detection using CT scan data. The liver was first separated using the watershed transform method, and the liver lesion was segmented using the GMM algorithm. Texture features were extracted and fed into a DNN model to automatically classify three types of liver tumors, including hemangioma, HCC, and metastatic carcinoma. The proposed model achieved a classification accuracy of 99.38% and a Jaccard index of 98.18%.

In 2020, Li et al. (108) developed a CAD system using ANN, SVM, and CNN models for diagnosing three types of HCC lesions, including nodular, diffuse, and massive. The experimental results demonstrated that the CNN model outperformed both the ANN and SVM models in classifying nodular and massive lesions, but not diffuse lesions.

In 2021, Mao et al. (25) developed a gradient boosting-based model using clinical parameters and CECT data for pathological grading of HCC. The combined model exhibited the best performance with an AUC of 0.8014 in the test set. Shi et al. (109) compared the performance of a DL-based three-phase CECT model with a four-phase CT protocol for distinguishing HCC from other FLLs. The DL-based three-phase CECT protocol without pre-contrast achieved a similar diagnostic accuracy (85.6%) to the four-phase CT protocol (83.3%). These findings suggest that omitting the pre-contrast phase might not compromise accuracy while reducing a patient’s radiation dose.

Several CNN-based models have been developed using CT data for diagnosing HCC. In 2018, Yasaka et al. (110) proposed a CNN model using three-phase CT for distinguishing malignant liver lesions from indeterminate and benign liver lesions. The proposed model achieved a median AUC of 0.92 in the test set. In 2019, Todoroki et al. (111) developed a CNN-based model using multiphasic CT images for detecting and classifying five types of FLLs. Ben-Cohen et al. (91) introduced a FCN architecture with sparsity-based false positive reduction for liver tumor detection, outperforming traditional models. By employing the FCN-4s model and sparsity-based fine-tuning, they successfully detected 94.7% of small lesions, surpassing the performance of the U-Net model.

In 2021, Zhou et al. (112) proposed a multi-modality and multi-scale CNN model for automatically detecting and classifying FLLs in multi-phasic CT. The model obtained an average test precision of 82.8%, recall of 93.4%, and F1-score of 87.8%. The model achieved average accuracies of 82.5% and 73.4% for the binary and six-class classification, respectively. In this study, the classification performance of the model was placed between a junior and senior physician’s evaluation. This preliminary study showed that this CNN-based model can accurately locate and classify FLLs, and could assist inexperienced physicians in reaching a diagnosis in clinical practice. Similarly, Ponnoprat et al. (113) constructed a two-step model based on CNN and SVM for distinguishing HCC and intrahepatic cholangiocarcinoma (ICC), and the model achieved a classification accuracy of 88%.

In 2021, Krishnan et al. (114) introduced a novel multi-level ensemble architecture for detecting and classifying HCC from other FLLs. This innovative approach highlights the potential of ensemble techniques in improving the specificity and sensitivity of liver cancer diagnosis using CT imaging.

In 2023, Manjunath et al. (115) developed a novel DL model using CT data to detect and classify liver tumors. The experimental results demonstrated that the proposed model improved accuracy, Dice similarity coefficient, and specificity compared to existing algorithms, emphasizing the continuous evolution of DL models for precise liver cancer diagnosis.

### 5.3 Prognostication of HCC

Numerous research groups have focused their efforts on the applications of AI models using CT and CECT images for the prognostication of HCC. **Table 4** provides a summary of the results from studies that evaluated AI-based CT models for HCC prognostication. Among these studies, Peng et al. (131) proposed a novel AI model based on conventional Machine Learning (cML) and DL methods. This model utilized CT data from 310 patients to predict TACE in patients with HCC. The experimental results demonstrated that the proposed model achieved AUC values of 0.995 and 0.994 in the training and testing datasets, respectively.

In 2021, Jiang et al. (81) developed a 3D CNN using CT data from 405 patients. This model was designed to predict Microvascular Invasion (MVI) in patients with HCC and obtained commendable AUC values of 0.98 and 0.906 in the training and testing datasets, respectively.

In 2022, Yang et al. (132) conducted an investigation of various AI models using CECT data from 283 patients. The aim was to predict MVI in patients with HCC. The experimental results revealed that the DL-based clinical-radiological model achieved the best performance with an accuracy of 96.47%, a sensitivity of 90.91%, a specificity of 97.30%, a precision of 89.4%, an F1 score of 87%, and an AUC of 0.909.

## 6 AI-based MRI methods

To date, the application of AI models in MRI for diagnosing HCC has not been extensively adopted. The development of MRI features poses technical challenges and incurs substantial costs, resulting in a scarcity of published studies with relatively small sample sizes. This section explores the progression of AI-based MRI models for the diagnosis of HCC.

### 6.1 Segmentation of liver and liver tumors

In recent years, a multitude of research groups have focused on the applications of AI models utilizing MRI data for the automated segmentation of the liver and liver tumors. **Table 5** encapsulates the AI-based MRI models recently developed for the segmentation of liver and liver tumors. Among the various studies, the most remarkable performance was delivered by Hossain et al. (139), who pioneered a cascaded network to address anatomical ambiguity. This model, which employs T1-weighted MRI data for liver segmentation, exhibited an impressive performance with a Dice coefficient of 0.9515, Intersection over Union (IoU) of 0.921, and an accuracy of 99.7%.

More recently, Gross et al. (140) developed a DCNN model using T1-weighted MRI data from 470 patients for liver segmentation. The results suggested that the proposed DCNN model achieved mean Dice values of 0.968, 0.966, and 0.928 in the training, validation, and public testing datasets, respectively.

### 6.2 Characterization of focal liver lesions

**Table 6** encapsulates the advancements in AI-based MRI models for diagnosing HCC. These models have shown promise in improving the detection and classification of FLLs, including HCC. In 2019, Hamm et al. (158) proposed a CNN model capable of classifying six types of FLLs, namely adenoma, cyst, Focal Nodular Hyperplasia (FNH), HCC, Intrahepatic Cholangiocarcinoma (ICC), and metastases. The model demonstrated an impressive overall accuracy of 92%, with sensitivity values spanning from 60% to 100%, and specificity values between 89% and 99%. This study highlighted the potential of DL in accurately identifying various types of FLLs.

Wang et al. (159) developed an interpretable DL model using MRI images. The model achieved a positive predictive rate of 76.5% and a sensitivity of 82.9% for classifying FLLs. The interpretability of this model enhances its clinical utility by offering insights into the decision-making process.

Trivizakis et al. (160) employed a 3D CNN model with Diffusion-Weighted Magnetic Resonance (DW-MR) data to classify primary and metastatic liver tumors. The model achieved an accuracy of 83%, underscoring the potential of DL in enhancing liver tumor recognition, particularly in datasets with limited size and disease specificity.

In 2020, Zhen et al. (161) pioneered several CNN models, including a distinctive model that utilizes unenhanced MR images for liver tumor diagnosis, thereby eliminating the need for contrast agent injection. This innovative approach demonstrated a performance on par with experienced radiologists, suggesting a potential reduction in patient discomfort and risks associated with contrast agents.

Kim et al. (162) introduced a CNN model that achieved an impressive AUC of 0.97, a sensitivity of 94%, and a specificity of 99% for HCC detection using a training dataset of 455 patients. In a validation dataset of 45 patients, the model maintained an AUC of 0.90, sensitivity of 87%, and specificity of 93% for HCC detection. This study underscored the capability of deep learning models in accurately identifying HCC, a critical step in early diagnosis and treatment planning.

Wu et al. (16) developed a DL model based on multiphase, contrast-enhanced MRI to differentiate between different grades of liver tumors for HCC diagnosis. The model utilized a CNN to classify the Liver Imaging Reporting and Data System tumor grades of liver lesions based on MRI data acquired at three-time points. The DL CNN model achieved high accuracy, sensitivity, precision, and AUC, providing valuable clinical guidance for differentiating between intermediate LR-3 liver lesions and more likely malignant LR-4/LR-5 lesions in HCC diagnosis.

In 2021, Wan et al. (163) proposed a CNN architecture based on multi-scale and multi-level fusion (MMF-CNN) for detecting liver lesions in MRI images. The model’s effectiveness was confirmed through comparative analysis with other DL models, emphasizing its potential to improve diagnostic accuracy and efficiency. The proposed MMF-CNN architecture is a promising approach to accurately and efficiently detect liver lesions in MRI images, which can significantly improve patient outcomes.

Oestmann et al. (164) presented a CNN model that employs multiphasic MR images to differentiate between HCC and non-HCC lesions. The model demonstrated high sensitivities and specificities for both lesion types, achieving 92.7% and 82.0% sensitivities for HCC and non-HCC lesions, respectively, and specificities of 82.0% for both HCC and non-HCC lesions. The research underscored the importance of accurately distinguishing between HCC and non-HCC lesions to guide appropriate treatment strategies for liver cancer patients.

Bousabarah et al. (148) proposed a CNN for detecting and segmenting HCC using multiphase contrast-enhanced MRI data. The model exhibited a promising performance with 73% and 75% sensitivities for validation and testing datasets, respectively. The performance evaluation compared the automatically detected lesions with manual segmentation. The mean Dice score values between the identified lesions using the CNN model and manual segmentations were 0.64 and 0.68 for the validation and testing datasets, respectively.

The advancements in CNN-based MRI models for diagnosing HCC have significantly enhanced the accuracy, efficiency, and precision of lesion classification and detection. From distinguishing different types of FLLs to detecting targeted HCC, these CNN-based models have showcased remarkable performance metrics and potential clinical utility. Further research and validation studies are essential to fully assess the capabilities of these models in clinical settings, paving the way for personalized and effective treatment strategies in liver cancer management.

### 6.3 Prognostication of HCC

A select number of research groups have ventured into the application of AI models and MRI-based data for HCC prognostication. **Table 7** encapsulates a summary of studies evaluating AI-based MRI models for this purpose.

In 2021, Gao et al. (168) scrutinized various AI models using T2-weighted MRI data from 225 patients to predict Microvascular Invasion (MVI) in patients with HCC. The H-DARnet model outshone others, achieving an accuracy of 82.6%, a sensitivity of 79.5%, a specificity of 73.8%, and an AUC of 0.775.

Wei et al. (187) investigated the fusion DL model and the Contrast-Dependent Learning Model (CDLM) using gadoxetic acid-enhanced MRI (EOB-MRI) data from 225 patients for predicting MVI in patients with HCC. Both models exhibited robust performance, with the Fusion DL model achieving an accuracy of 89.4%, a sensitivity of 78.1%, a specificity of 95.3%, and an AUC of 0.93. The CDLM model achieved an accuracy of 92.4%, a sensitivity of 93.9%, a specificity of 91.6%, and an AUC of 0.962 in the training dataset.

In 2023, Chen et al. (169) explored four models (KNN, SVM, Lasso, and DNN) using T2-weighted MRI data from 144 patients for predicting Transarterial Chemoembolization (TACE) outcomes in patients with HCC. Among these, the Lasso model achieved the best performance.

These studies underscore the potential of AI models in conjunction with MRI data for predicting HCC, demonstrating promising results in terms of accuracy, sensitivity, specificity, and AUC. Further research in this area could catalyze significant advancements in the early detection and treatment of HCC.

## 7 AI-based multi-modal techniques

AI-based multi-modal techniques are swiftly ascending to prominence in the realm of medical imaging, attributed to its extraordinary ability to amplify diagnostic accuracy and forecast outcomes. AI-based multi-modal model integrates multiple modalities, such as medical imaging data, Electronic Health Records (EHR) and clinical parameters, thereby substantially enhancing the efficacy of AI algorithms. AI-based multi-modal models have proven successful in predicting treatment responses, evaluating survival rates, and staging a multitude of diseases. Such techniques have been deployed in a plethora of studies pertaining to liver imaging applications, yielding encouraging results. The continued exploration and refinement of these techniques hold great promise for the future of medical imaging and patient care.

### 7.1 Segmentation of liver and liver tumors

**Table 8** encapsulates a summary of studies that evaluate AI-based multi-modal models for the segmentation of liver and liver tumors. Among the various studies, the most remarkable performance was demonstrated by Hille et al. (173). They explored the SWTR-Unet model using a combination of 61,440 MRI images and 189,600 CT images for the segmentation of both the liver and hepatic lesions. The proposed multi-modal model achieved Dice coefficients of 0.98 and 0.81 for the segmentation of the liver and hepatic lesions, respectively.

### 7.2 Diagnosis of HCC

AI-based multi-modal models offer a comprehensive and robust approach to HCC diagnosis, enabling disease prediction, classification, treatment response prediction, survival rate determination, and disease staging. The outcomes of studies evaluating AI-based multi-modal models for HCC diagnosis are summarized in **Table 9**.

In 2020, Menegotto et al. (176) utilized a DCNN for HCC diagnosis, incorporating CT data and various EHR parameters. These parameters encompassed demographic factors, clinical history, laboratory test results, and other pertinent medical information. The model achieved accurate HCC diagnosis by considering 20 unique EHR parameters, highlighting the potential of integrating diverse clinical data for enhanced disease identification. Subsequently, they (177) developed an Xception CNN model using CT data and EHR parameters for HCC diagnosis. This method accurately detected HCC, demonstrating the potential of combining various modalities for improved HCC identification.

Zhen et al. (161) developed a multi-modal model that combines Google’s Inception-ResNetV2 CNN with an autoencoder neural network. This model was used to diagnose HCC using MRI data and clinical parameters, including age, gender, tumor markers, liver function, and other relevant factors. The study confirmed the potential of combining medical imaging and clinical data to improve HCC diagnosis, emphasizing the importance of such techniques in enhancing healthcare outcomes.

In 2021, Gao et al. (118) employed a multi-modal model based on the VGG16 architecture to detect HCC in CT images. The study aimed to determine the model’s accuracy in detecting HCC by incorporating eight EHR parameters, including age, gender, platelet count, bilirubin levels, tumor markers, and hepatitis B virus status. The research findings demonstrated the capacity of multi-modal DL to accurately identify HCC. This study underscores the potential of ML algorithms in assisting the early detection and diagnosis of HCC, which may lead to improved patient outcomes. Li et al. (179) investigated a ML-based multi-modal model using three-phase CEUS data from 266 patients and a radiologist’s score for evaluating the diagnostic accuracy when differentiating between atypical Hepatocellular Carcinoma (aHCC) and Focal Nodular Hyperplasia (FNH). The proposed model achieved the highest AUC of 0.93 in aHCC and FNH differentiation.

In 2022, Liu et al. (180) proposed a DL model to detect malignancy by combining clinical parameters and CEUS data from 303 patients. The model achieved the best performance with AUC values of 0.969 and 0.957 and accuracies of 96% and 94% in the IntraVenous (IV) and ExtraVenous (EV) groups, respectively. Further research is necessary to identify the optimal combination of modalities and variables for specific medical tasks. The development of standardized protocols and datasets is critical to facilitate the comparison and reproducibility of multi-modal AI models in medical image analysis.

### 7.3 Prognostication of HCC

A multitude of studies have explored the use of AI-based multi-modal models for prognostication of HCC. The insights from these studies are compiled in **Table 10**. Among these, a significant contribution was made by Sun et al. (183), who implemented a hybrid model combining GhostNet and CNN models. This integrated model leveraged CT data and clinical parameters to predict the response of TACE treatment in HCC patients. The proposed method exhibited remarkable performance, achieving an accuracy of 98% and an AUC of 0.98. This model demonstrated its potential in predicting TACE treatment responses, thereby assisting healthcare providers in devising personalized treatment plans and making informed decisions. This approach shows promise in improving patient outcomes and raising the bar in clinical practice.

## 8 Challenges and future directions

In the past decade, AI models’ application in medical imaging for HCC diagnosis and prediction has emerged as a significant research area. While individual medical imaging methods such as US, CT, and MRI have been explored (205–208), there is a lack of comprehensive reviews focusing on AI-based models using both single and multi-modal modalities. This study aims to fill that gap, reviewing AI models developed for HCC diagnosis and prediction using both single and multi-modal methods from January 2010 to March 2024.

Despite AI-based diagnostic models not significantly improving overall diagnostic accuracy for pathologists, they have shown increased precision within specific subgroups. However, several challenges must be addressed before integrating these models into clinical workflows. The efficacy of AI models depends on both the models’ accuracy and the quality of the datasets used. Factors such as biases, mislabeling, lack of standardization, and missing data can undermine these datasets. Overfitting and spectrum biases are prevalent issues in AI-based medical imaging models. Therefore, the need for standardized methods for AI-based data analysis and comprehensive strategies to tackle missing data is evident.

AI tools intended for medical applications could be categorized as medical devices and must adhere to pertinent regulations. Both the FDA and the European Commission have initiated plans to tackle this issue. Intellectual property concerns, particularly those associated with post-marketing modifications, could pose safety risks. The performance of AI models is intimately linked to the training dataset. The importance of large datasets is paramount, and the promotion of data sharing is necessary, which brings forth ethical and privacy considerations. The clinical performance of AI and the requirement for post-approval validation are significant issues. The development of explainable AI models is vital for securing clinicians’ trust and reliance on AI-based CAD systems. Customized prospective clinical trials are indispensable to fully comprehend the role of AI in HCC management.

Looking ahead, the integration of AI in HCC management presents an exciting frontier in medical science. As we continue to refine AI models and address the challenges, we move closer to a future where AI plays a pivotal role in personalized patient care. The potential of AI to analyze vast amounts of data and make precise predictions can lead to early detection and more effective treatment strategies for HCC. This not only improves patient outcomes but also paves the way for a new era in healthcare, where technology and human expertise work hand in hand for the betterment of patient care.

Several strategies are essential for the future of AI in HCC diagnosis and prediction. First, the development of standardized methods for AI-based data analysis and comprehensive strategies to handle missing data are crucial. Second, universal approaches to handle missing data and improve data quality are vital for enhancing the robustness and reliability of DL-based diagnostic tools. Promoting data sharing initiatives can facilitate the availability of large, diverse datasets necessary for training and validating DL models.

In addition to the aforementioned strategies, the exploration of advanced technologies such as transfer learning can further enhance the role of AI in HCC diagnosis and prediction. This technology can adapt pre-trained DL models to new tasks with limited labeled data. This addresses the challenge of acquiring extensive datasets in medical imaging, a common hurdle in the healthcare sector. Federated Learning (FL) is emerging as a transformative trend in healthcare. It enables a collaborative approach to ML development across multiple institutions, eliminating the need for direct data sharing. This innovative method involves the exchange of model parameters only, thereby ensuring the privacy of individual datasets. In the context of liver cancer, where patient data is both sensitive and heavily regulated, FL offers a unique advantage. It allows for the integration of fragmented healthcare data sources while preserving privacy. This enhances the scope and accuracy of ML models, making them more effective and reliable. As such, FL is poised to become an invaluable tool for future research and clinical implementation in liver cancer treatment. It offers the potential to significantly advance patient care, marking a new era in the field of liver cancer treatment.

The development of explainable AI models is another critical step towards earning the trust and reliance of clinicians on AI-based CAD systems. The synergy of researchers, clinicians, and policymakers is a cornerstone in propelling innovation and setting the gold standard for the application of AI techniques in liver cancer care. A comprehensive approach is required to augment AI techniques for HCC diagnosis and management. This involves addressing key aspects such as interpretability, accuracy, data integration, ethical considerations, and validation processes. By tackling these areas, we can tap into the full potential of AI technology, leading to a revolution in HCC diagnosis and prediction. Customized prospective clinical trials are paramount to gain a complete understanding of the role of AI in HCC management. Regulatory bodies like the FDA and the European Commission have kick-started plans to address the regulatory compliance of AI-based diagnostic tools. These plans demand further development and implementation. The challenges and future directions underscore the intricacy of incorporating AI in HCC diagnosis and prediction. However, with persistent research and development, AI holds the promise to bring about a paradigm shift in this field.

## 9 Conclusions

This paper offers an exhaustive exploration of AI-driven models for the diagnosis and prediction of HCC, leveraging both medical imaging data and additional clinical information. The potential of AI-based methodologies in diagnosing HCC is vast, yet several hurdles need to be overcome before they can be seamlessly incorporated into clinical workflows to enhance patient diagnosis and treatment outcomes. Despite the presence of challenges such as data quality, model overfitting, regulatory compliance, and the necessity for explainable AI models, the potential advantages are considerable. AI models have the capacity to augment precision within specific patient subgroups. Furthermore, the development of standardized methods for data analysis can significantly bolster the robustness and reliability of these tools. Navigating these intricacies, it becomes evident that a multi-pronged strategy is essential to fully harness the transformative power of AI technology in revolutionizing HCC diagnosis and treatment. With ongoing research and development, AI stands poised to usher in a paradigm shift in the field of HCC diagnosis and prediction, ultimately leading to enhanced patient outcomes and heralding a new epoch in healthcare.
